# Effects of *lng* Mutations on LngA Expression, Processing, and CS21 Assembly in Enterotoxigenic *Escherichia coli* E9034A

**DOI:** 10.3389/fmicb.2016.01201

**Published:** 2016-08-03

**Authors:** Zeus Saldaña-Ahuactzi, Gerardo E. Rodea, Ariadnna Cruz-Córdova, Viridiana Rodríguez-Ramírez, Karina Espinosa-Mazariego, Martín A. González-Montalvo, Sara A. Ochoa, Bertha González-Pedrajo, Carlos A. Eslava-Campos, Edgar O. López-Villegas, Rigoberto Hernández-Castro, José Arellano-Galindo, Genaro Patiño-López, Juan Xicohtencatl-Cortes

**Affiliations:** ^1^Laboratorio de Investigación en Bacteriología Intestinal, Hospital Infantil de México Federico GómezCiudad de México, Mexico; ^2^Instituto de Fisiología Celular at the Universidad Nacional Autónoma de MéxicoCiudad de México, Mexico; ^3^Departamento de Genética Molecular, Instituto de Fisiología Celular, Universidad Nacional Autónoma de MéxicoCiudad de México, Mexico; ^4^Departamento de Salud Pública, Facultad de Medicina, Universidad Nacional Autónoma de MéxicoCiudad de México, Mexico; ^5^Laboratorio Central de Microscopía, Departamento de Investigación-SEPI, Instituto Politecnico NacionalCiudad de México, Mexico; ^6^Departamento de Ecología de Agentes Patógenos, Hospital General “Dr. Manuel Gea González”Ciudad de México, Mexico; ^7^Departamento de Infectología, Hospital Infantil de México Federico GómezCiudad de México, Mexico; ^8^Laboratorio de Investigación en Inmunología y Proteómica, Hospital Infantil de México Federico GómezCiudad de México, Mexico

**Keywords:** ETEC, CS21, biogenesis, type IV pilus, adherence to intestinal cells, pilus

## Abstract

Enterotoxigenic *Escherichia coli* (ETEC) is a major cause of morbidity in children under 5 years of age in low- and middle-income countries and a leading cause of traveler's diarrhea worldwide. The ability of ETEC to colonize the intestinal epithelium is mediated by fimbrial adhesins, such as CS21 (Longus). This adhesin is a type IVb pilus involved in adherence to intestinal cells *in vitro* and bacterial self-aggregation. Fourteen open reading frames have been proposed to be involved in CS21 assembly, hitherto only the *lngA* and *lngB* genes, coding for the major (LngA) and minor (LngB) structural subunit, have been characterized. In this study, we investigated the role of the LngA, LngB, LngC, LngD, LngH, and LngP proteins in the assembly of CS21 in ETEC strain E9034A. The deletion of the *lngA, lngB, lngC, lngD, lngH*, or *lngP* genes, abolished CS21 assembly in ETEC strain E9034A and the adherence to HT-29 cells was reduced 90%, compared to wild-type strain. Subcellular localization prediction of CS21 proteins was similar to other well-known type IV pili homologs. We showed that LngP is the prepilin peptidase of LngA, and that ETEC strain E9034A has another peptidase capable of processing LngA, although with less efficiency. Additionally, we present immuno-electron microscopy images to show that the LngB protein could be localized at the tip of CS21. In conclusion, our results demonstrate that the LngA, LngB, LngC, LngD, LngH, and LngP proteins are essential for CS21 assembly, as well as for bacterial aggregation and adherence to HT-29 cells.

## Introduction

Enterotoxigenic *Escherichia coli* (ETEC) remains as a major bacterial pathogen associated to high morbidity, mainly in children less than 5 years of age in low- and middle-income countries. The mortality of ETEC in 2010 was estimated to be of 120,800 deaths (Lozano et al., 2013). ETEC is also a leading cause of diarrheal illness in travelers to endemic countries (Nataro and Kaper, 1998; Kaper et al., 2004). ETEC possess two enterotoxins: thermo-labile (LT) and thermo-stable (ST), which are responsible for the secretory diarrhea (Nataro and Kaper, 1998). The colonization factors (CFs) are indispensable for ETEC virulence, and are responsible for intestinal epithelium adherence and colonization (Gaastra and Svennerholm, 1996). More than 20 CFs have been identified, however, only eight (CFA/I, CS1, CS2, CS3, CS4, CS5, CS6, and CS21) are the most prevalent worldwide (Gaastra and Svennerholm, 1996; Isidean et al., 2011). CS21, also called longus, is a class b type IV pilus which is encoded in a 14 kb *lng* gene cluster located in a large virulence plasmid (50–90 kb) (Girón et al., 1994; Gomez-Duarte et al., 2007). Type IV pilus have been classified into two classes: IVa and IVb. Type IVa pilus assembly requires around 40 genes products encoded in different parts of the genome; whereas type IVb system comprises about a dozen genes (between 11 and 14), encoded within the pilus operon (Strom and Lory, 1993; Roux et al., 2012). Girón et al. (1994) reported for the first time that CS21 structural gene (*lngA*) is encoded in a large plasmid, and that a 5-kb Bam HI restriction fragment from this plasmid was sufficient for pilus assembly in an *Escherichia coli (E. coli)* strain K-12 (DH5αF′IQ). Gomez-Duarte et al. (2007) reported a cluster of 14 genes (*lngR, lngS, lngT, lngA, lngB, lngC, lngD, lngE, lngF, lngG, lngH, lngI, lngJ*, and *lngP*) of 13.93 kb located in tandem that might be required for expression of CS21.

The CS21 filament is composed of a 22-kDa monomer (LngA) which shares considerable N-terminal sequence similarity with CofA of ETEC, TcpA of *Vibrio cholerae* and BfpA of enteropathogenic *E. coli* (EPEC) (Taylor et al., 1987; Girón et al., 1997). The *lngA* gene codes for an immature LngA protein (prepilin), and requires processing by a prepilin peptidase, before of its translocation and oligomerization. The processing includes signal sequence removal and first residue (methionine) methylation, leading to a mature pilin (Hobbs and Mattick, 1993; Strom and Lory, 1993).

The biogenesis of type IV pilus involves oligomerization and secretion of the pilin subunit by a mechanism that requires a set of proteins, which are part of an assembly and export apparatus. The assembly of type IV pili also requires a nucleotide-binding protein that provides energy, a polytopic inner membrane protein, a prepilin peptidase, and accessory proteins (Mattick, 2002). Several proteins involved in pilus biogenesis of the Bundle-Forming Pilus (BFP) of EPEC, Toxin Corregulated Pilus (TCP) of *V. cholerae*, and Colonization Factor Antigen III (CFA/III) of ETEC, are homologous with proteins encoded in the *lng* cluster (Gomez-Duarte et al., 2007; Roux et al., 2012). For example, LngD has two homologs, BfpB and TcpC, that are multimeric outer membrane lipoproteins through which the pilin subunits BfpA and TcpA are extruded (Ogierman and Manning, 1992; Ramer et al., 1996). The high similarity between CS21 and CFA/III biosynthetic genes indicates that CS21 and CFA/III are paralogous (Gomez-Duarte et al., 2007).

LngA, LngB, LngJ, and LngP have more than 60% of identity with CofA, CofB, CofJ, and CofP, respectively (Gomez-Duarte et al., 2007). The latter proteins have been previously characterized. CofA is the major pilin subunit of CFA/III (Taniguchi et al., 2001), and CofB the minor pilin subunit, which has been proposed to initiate pilus assembly (Kolappan et al., 2015). CofJ is a soluble protein secreted via the CFA/III system and has been proposed to be a putative adhesin of ETEC (Yuen et al., 2013). CofP has a prepilin peptidase function (Taniguchi et al., 1999).

CS21 induces bacterial self-aggregation, which protects ETEC against antimicrobial agents *in vitro* (Clavijo et al., 2010). The role of CS21 in human colonic cells colonization has been studied *in vitro* by Mazariego-Espinosa et al. (2010); and recently, Guevara et al. (2013) demonstrated the role of CS21 in the pathogenesis of ETEC *in vivo* using a neonatal mice challenge infection model. However, the proteins involved in CS21 assembly have not been fully described. The purpose of this study was to determine the effect of the deletion of six *lng* (*lngA, lngB, lngC, lngD, lngH*, and *lngP*) genes in the ETEC strain E9034A on CS21 assembly. These genes were chosen based on its putative function with homologous proteins of other type IV pili. Here we report that deletions of the *lngA, lngB, lngC, lngD, lngH*, and *lngP* genes affect CS21 assembly, bacterial self-aggregation, and adherence to HT-29 cells.

## Materials and methods

### Bacterial strains and culture media

The strains and plasmids used in this study are listed in Table 1. All the strains were stored in Luria-Bertani (LB) broth (Difco, NJ, USA) with 20% glycerol at −70°C. Strains were grown on LB agar or in LB broth at 30, 37, or 42°C depending on the assay. For CS21 expression, Pleuropneumonia-Like Organisms (PPLO) broth (BD, Difco, NJ, USA) or terrific broth (TB) (Amresco LLC, OH, USA) media was used. When required, kanamycin (50 μg/ml), ampicillin (100 μg/ml) and/or chloramphenicol (50 μg/ml) antibiotics were added to the media.

**Table 1:** **Strains and plasmids used in this study**.

**Strain or plasmid**	**Description**	**References or source**
E9034A	Wild-type ETEC (O8:H9, SL^+^/LT^+^)	Levine et al., 1984
DH5α	*E. coli* K-12 *supE44*Δ*lacU169* F80 *lacZ* ΔM15 *hsdR17 recA1 endA1 gyrA96 thi-1 relA1*	Lab collection
TOP10	One Shot® TOP10 *E. coli*, F- *mcrA* Δ(mrr-hsdRMS-mcrBC) φ80*lacZ*ΔM15 Δ*lacX*74 *nupG recA1 araD139* Δ(*ara-leu*) 7697 *galE15 galK16 rpsL* (Str^R^) *endA1 λ*−	Lab collection
BL21 (DE3)	*E. coli* K-12 *fhuA2 [lon] ompT gal (λ DE3) [dcm] ΔhsdS* λ *DE3 = λ sBamHIo ΔEcoRI-B int::(lacI::PlacUV5::T7 gene1) i21 Δnin5*	Lab collection
E9034AΔ*lngA::km*	E9034A with a non-polar insertional mutation in *lngA* and kanamycin resistance	Cruz-Córdova et al., 2014
E9034AΔ*lngB::cm*	E9034A with a non-polar insertional mutation in *lngB* and chloramphenicol resistance	This study
E9034AΔ*lngC::cm*	E9034A with a non-polar insertional mutation in *lngC* and chloramphenicol resistance	This study
E9034AΔ*lngD::cm*	E9034A with a non-polar insertional mutation in *lngD* and chloramphenicol resistance	This study
E9034AΔ*lngH::cm*	E9034A with a non-polar insertional mutation in *lngH* and chloramphenicol resistance	This study
E9034AΔ*lngP::cm*	E9034A with a non-polar insertional mutation in *lngP* and chloramphenicol resistance	This study
E9034AΔ*lngR::km*	E9034A with a non-polar insertional mutation in *lngR* and kanamycin resistance	Unpublished data
**Plasmids**		
pKD46	λ Red recombinase system plasmid	Datsenko and Wanner, 2000
pKD3	Cm^r^ cassette template plasmid	Datsenko and Wanner, 2000
pKD4	Km^r^ cassette template plasmid	Datsenko and Wanner, 2000
pUC18	High-copy-number cloning vector	Lab collection
pUC19	High-copy-number cloning vector	Lab collection
pBAD-Topo	Low-copy-number expression vector	Invitrogen
pJET1.2/blunt	Low-copy number cloning vector	ThermoFisher
pLATE31	Bacterial expression vectors (Ampr)	ThermoFisher Scientific
	His-tag in C-terminal	
pUClngA	*lngA* in pUC18 (Amp^r^)	This study
pUClngB	*lngB* in pUC19 (Amp^r^)	This study
pUClngC	*lngC* in pUC19 (Amp^r^)	This study
pUClngD	*lngD* in pUC19 (Amp^r^)	This study
pBADlngH	*lngH* in pBAD-Topo (Amp^r^)	This study
pUClngP	*lngP* in pUC19 (Amp^r^)	This study
pLATE31-lngC	*lngC* in pLATE31	This study
pLATE31-lngB	*lngB* in pLATE31	This study

*Km^r^, kanamycin resistance; Amp^r^, ampicillin resistance; Cm^r^, chloramphenicol resistance*.

### Construction of isogenic mutants

Non-polar deletion mutants in *lngB, lngC, lngD, lngH*, and *lngP* genes were generated by the λ-Red recombinase method (Datsenko and Wanner, 2000). The primers employed for DNA amplification are listed in Table 2. Primers flanking the *lngB, lngC, lngD, lngH*, and *lngP* genes were used to confirm the gene deletion and replacement by PCR (Table 2). The mutant strains were complemented by the addition of a wild-type copy of the mutant gene in *trans* on a high-copy-number vector (Table 1). Empty plasmids were transformed into each mutant to verify that complementation was due to the presence of the wild-type gene.

**Table 2:** **List of primers used in this study**.

**Primer**	**Sequence**	**Use**
LngA-FC	5′ 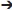 TCGCCATGGGGATCCAATTACGTAAACAACGT-3′	Cloning of *lngA* in pUC18
LngA-RC	5′ 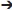 TTGAAGCTTTTAACGGCTACCTAAAGTAATTG-3′	Cloning of *lngA* in pUC18
LngA-FP	5′ 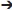 TGCGGATCCGTGATCTGAAGAAAAATAA-3′	Cloning of *lngA* in pJET1.2/blunt
LngA-RP	5' TGTGAGAAGGTACTAGCCTATCATATT-3′	Cloning of *lngA* in pJET1.2/blunt
LngB-FM	5′ 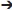 CACAGAGACAATTTTATGAAAATGAGAGGCTTCGTGTAGGCTGGAGCTGCTTC-3′	Mutagenesis
LngB-RM	5′ 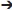 CATTTTATCCTCTCCATTACATTAGGTTTGTGGTTCTGTACTATATGAATATCCTCCTTAG-3′	Mutagenesis
LngB-FS	5' GTAGAGCTCAGGTAAACTCAATTACTTTAGGTAGCCGT-3′	Screening mutants
LngB-RS	5′ 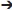 TGCAAGCTTCAGGAGATGCCAGTGCACTCG-3′	Screening mutants
FlngBALL	5′ 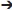 AGAAGGAGATATAACTATGAAAATGAGAGGCTTCACACTTCTGGAGATGATTATCACTCTCGC-3′	Cloning of *lngB* in pLATE31
RlngBALL	5′ 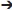 GTGGTGGTGATGGTGATGGCCGGTTTGTGGTTCTGTACTGCACCAGGTTGTGATA-3′	Cloning of *lngB* in pLATE31
LngC-FM	5′ 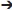 AATGGAGAGGATAAAATGAGAGCAAAATGGGTGTAGGCTGGAGCTGCTTC-3′	Mutagenesis
LngC-RM	5′ACTTCATATTCTTATACCTGACATTCATTGAGAGTCGATAACCAGTACATATGAATATCCTCCTTAG-3′	Mutagenesis
LngC-FS	5′ 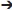 CAACCATGGGGATCCCAGAACCACAAACCTAATG-3′	Screening mutants
LngC-RS	5′ 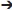 TGATGAGAATTCCTGCAATGCCAATAAAACTAACATC-3′	Screening mutants
FlngCALL	5′ 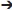 AGAAGGAGATATAACTATGAGAGCAAAATGGGGTGTTTTTTTCTTCCTTAGTATTCTTTCTTCGAG-3′	Cloning of *lngC* in pLATE31
RlngCALL	5′ 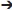 GTGGTGGTGATGGTGATGGCCTTGAGAGTCGATAACCAGTACGTTGTTTC-3′	Cloning of *lngC* in pLATE31
LngD-FM	5′ 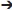 ACTGGTTATCGACTCTCAATGAATGTCAGGTATAAGAATGTGTAGGCTGGAGCTGCTTC-3′	Mutagenesis
LngD-RM	5′ 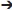 CGATAATAGTCAATACCTACAACCATTGCGATGGTATTATCAATATGAATATCCTCCTTAG-3′	Mutagenesis
LngD-FS	5′ 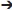 GTCCCATGGGGATCCGTTTTCTTCAGAACAATAT-3′	Screening mutants
LngD-RS	5′ 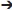 CCATAAGAGCTCCAGCGCAATTTTTTCATC-3′	Screening mutants
LngH-FM	5′ 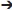 CTTGTACATAAAGTTAAAATGACGGGCCTTGCAGTCGTTGTGTAGGCTGGAGCTGCTTC-3′	Mutagenesis
LngH-RM	5′ 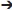 CAAAACTGATAGAGATAAATACGTTGTTTTTTATTGAATTTTTTCAAGTAATACCTCATATGAATATCCTCCTTAG-3′	Mutagenesis
LngH-FS	5′ 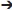 AGAGAATTCCCGGGAAAGTACAGGCTG-3′	Screening mutants
LngH-RS	5′ 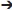 GAGTCATAGATCGGTAATCCTGAAAGCTTCAT-3′	Screening mutants
LngP-FM	5′ 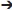 ATGTATGTTGAAATCGGCGTTTTCTTTTTTTTATTCATTACAGTGTAGGCTGGAGCTGCTTC-3′	Mutagenesis
LngP-RM	5′ 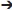 ATCTCTATGCATTTCTCATAGAAATAAGAGAAAGTATTCATAAACATATGAATATCCTCCTTAG-3′	Mutagenesis
LngP-FS	5′ 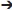 GTTGGATCCAGATTTAGTGGGCCT-3′	Screening mutants
LngP-RS	5′ 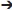 GATGTCGACGAGCTCTATAGATATTAAATCTCTAT-3′	Screening mutants

### Molecular and genetic techniques

All oligonucleotides used for PCR were obtained from Integrated DNA Technologies, Inc (IDT, CA, USA) and are listed in Table 2. PCR, restriction endonuclease digestion, ligation, transformation, plasmid DNA preparations, and DNA electrophoresis were performed using standard techniques (Sambrook and Russell, 2001). PCR amplifications were performed with PCR Master Mix 2x (Promega Corporation, WI, USA) and Platinum® *Taq* DNA Polymerase High Fidelity (Invitrogen, USA). Restriction enzymes were purchased from Promega (Promega Corporation, WI, USA) and were used according to the manufacturer's recommendations.

### Cloning of the *lngB* and *lngC* genes in the pLATE31 expression plasmid

Primer design, amplification, cloning and expression of *lngB* and *lng* genes in the pLATE31 plasmid were conducted according to the aLICator Ligation Independent Cloning and Expression System (Thermo Scientific, CA, USA). pLATE31 and the recombinant expression plasmid pLATE31-lngB and pLATE31-lngC were transformed into *E. coli* BL21 (DE3). For the expression and detection of LngB and LngC, *E. coli* was cultured in LB medium at 37°C, and induced with 1 mM IPTG for 5 h. LngB and LngC His-Tag recombinant proteins were purified from 200 ml of induced cultures media. The cultures were centrifuged at 3500 × *g*, and the pellets were resuspended in 20 ml of pH 8.2 lysis buffer (8M Urea, 100 mM NaH_2_PO_4_, 10 mM Tris-HCl, and 10 mM imidazole) and sonicated for 30 min at 40 kHz, using 10 cycles of 1 min pulse and 2 min of cooldown. The sonicated samples were centrifuged at 3500 × *g*, and the His-tag recombinant proteins from the clear supernatants were captured in an affinity chromatography column of Ni-NTA Agarose (Qiagen, Hilden, Germany), washed with 60 ml of lysis buffer (pH 6.5), and eluted with 10 ml of lysis buffer (pH 4.0). The eluted fractions containing the recombinant proteins were refolded by dialysis and maintained in phosphate-buffered saline (PBS) pH 7.0. Anti His-Tag monoclonal antibodies were used to detect the expression of recombinant proteins by immunoblotting.

### Antibodies production

Rabbit anti-CS21, anti-LngB, and anti-LngC sera were produced by immunization of New Zealand white rabbits with either purified CS21 obtained from the ETEC strain E9034AΔ*lngR::km* (Saldaña et al., unpublished data) or C-terminal His-Tag LngB and LngC purified proteins. Rabbits were immunized every 2 weeks, and emulsions in complete Freund's adjuvant (1 dose with 1 μg of antigen in 500 μl of PBS + 500 μl of adjuvant) or incomplete Freund's adjuvant (3 doses with 0.5 μg of antigen in 500 μl of PBS + 500 μl of adjuvant) were subcutaneously administered. The antisera obtained were adsorbed 8 times against E9034AΔ*lngA*, E9034AΔ*lngB*, and E9034AΔ*lngC* strains respectively, to remove nonspecific antibodies and increase the specificity. The antisera were used in Western blot (WB), immunofluorescence (IF), and immunogold assays, as described below.

### Adherence assays to HT-29 cells

Adherence assays were carried out in 24-well tissue culture plates (Corning, NJ, USA), with or without 12 mm round coverslips (Bellco Glass, NJ, USA). Colon adenocarcinome HT-29 (ATCC HTB-38, VA, USA) cell monolayers were used as previously described (Saldana et al., 2009). Briefly, approximately 5 × 10^6^ colony forming units (CFU) grown overnight in PPLO were added to HT-29 monolayers at 80% confluence (1 × 10^5^ cells), which were then incubated at 37°C for 6 h in a 5% CO_2_ atmosphere. Infected monolayers were washed with PBS 1X and then lysed with 0.1% Triton X-100 (Amresco Bioscience, OH, USA) in PBS 1X, and the bacteria were quantified by plate counts. Adhesion assays were performed in triplicate on three different days to obtain an average of the data expressed as CFU/ml. The standard deviations were calculated from all the results and represented as error bars in the graphs. Samples with coverslips were fixed with 2% formaldehyde in PBS 1X for Giemsa staining or for immunofluorescence microscopy (IFM) as previously described by Saldana et al. (2009).

### Immunofluorescence assay

The assay was carried out to visualize CS21 assembly by E9034A ETEC strain and isogenic mutants attached to HT-29 cells. Samples were incubated for 5 min at room temperature with 0.1% Triton X-100 in PBS, followed by a 30 min incubation with RNase (Sigma-Aldrich-Co. LLC, MO, USA) diluted 1:5000 in PBS 1X with 10% horse serum (PBS-HS) at 37°C. Cells were then incubated with anti-CS21 serum diluted 1:2000 in PBS-HS, followed by goat anti-rabbit IgG antibodies conjugated with Alexa Fluor® 488 (Abcam, Cambridge, UK) diluted 1:2500 in PBS-HS and incubated during 1 h at 37°C. Cells were washed 3 times with 1 ml of PBS after each step. Coverslips were mounted on glass slides with 3 μl of DAPI (Merck Millipore, USA) or propidium iodide (Sigma-Aldrich-Co. LLC, MO, USA). Samples were visualized under an Axio Imager 2 Research Microscope (Zeiss, GER). Confocal microscopy images were taken in a Leica TCS SP8x microscope with a 63x oil immersion objective and digital zoom of 5x. The samples were analyzed with the Leica Application Suite software; Advanced Fluorescence Lite version 2.6.3 build 8173 (LAS AF Lite; Leica Microsystems, GER).

### Transmission electron microscopy (TEM) and immuno-electron microscopy (IEM)

Bacterial cultures drops (10 μl) were placed onto formvar-coated 300 mesh copper grids and adsorbed for 5 min, fluid excess was wiped with filter paper. Subsequently, 4 min negative staining was performed with 6 μl of 1% phosphotungstic acid (pH 7.4) (EMS, PA, USA); any excess was removed using filter paper. The grids were rinsed with two drops of distilled water and finally were air-dried. The samples were examined by TEM using a JEM-1010 microscope (JEOL, Tokyo, Japan). IEM studies were performed to confirm the presence or absence of CS21 and LngB on E9034 strains and the isogenic mutants. The copper grids covered with the bacterial cultures were incubated for 1 h with anti-CS21 or anti-LngB antibodies (diluted 1:10 in PBS containing 10% BSA). The samples were rinsed with a drop of PBS three times, followed by 1 h incubation with a goat anti-rabbit IgG H&L conjugated to 10 nm gold particles (Sigma-Aldrich-Co, MO, USA) and rinsed again as previously described. Subsequently, a negative staining was performed as previously described.

### Protein electrophoresis and western-blot

For the analysis of whole-cell extracts, 5 ml of PPLO cultures were grown overnight with shaking at 37°C or supernatants from adherence assay were adjusted to 1 ml of culture with an optical density (OD_600_) of 1.0, harvested by centrifugation and lysed by heating at 95°C for 5 min in 200 μl of loading buffer (Laemmli, 1970). Whole-cell extracts from equivalent cell numbers were resolved by SDS-PAGE and endogen chaperone DnaK was used as a loading control. Twenty-five microliter aliquots were loaded into SDS-PAGE gels. Gels were run at 100 V for 2 h at room temperature. For the WB, proteins separated by electrophoresis were transferred to PDVF membranes at 21 V for 1 h. The blots were blocked with PBS containing 0.1% (vol/vol) Tween 20 and 5% milk. Blocked membranes were reacted for 1 h with anti-CS21 or anti-DnaK antibodies (MBL International, MA, USA) in PBS-Tween 0.1%, washed 3 times with PBS-Tween 0.1% and incubated for 1 h with goat anti-rabbit IgG conjugated with horseradish peroxidase (Sigma-Aldrich-Co, MO, USA). The membranes were washed and revealed by chemiluminescence (ECL) (Amersham Life Science, Ill, USA).

### Self-aggregation assay

Wild-type and isogenic mutants were grown overnight in 5 ml of TB at 37°C. A 1:100 dilution of each culture was incubated in flat-bottom 24-well tissue culture plates (Corning, NY, USA) at 37°C for 4 h. The bacterial self-aggregation phenotype consists of bacterial clumping. The clumps were visualized directly on the culture plates using an inverted light microscope (Olympus, Center Valley, PA; Clavijo et al., 2010). Triplicates of the assays were performed at three different times. ETEC E9034A and *E. coli* DH5α strains were used as positive and negative controls, respectively. Bacterial-bacterial aggregation mediated by CS21 causes sedimentation and media clearance. Quantitative analysis was accomplished by measuring the absorbance at 600 nm of the supernatant cultures without disturbing the aggregates.

### Bioinformatic analysis

Bacterial protein subcellular localization was predicted using the following servers: CELLO v.2.5, from the Molecular Bioinformatics Center (http://cello.life.nctu.edu.tw/), which is an amino acid composition-based method; PSLpred from the Bioinformatics center Institute of Microbial Technology (http://www.imtech.res.in/raghava/pslpred/); and PSORTb version 3.0.2 from the Brinkman Laboratory, Simon Fraser University (http://www.psort.org/psortb/). Bioinformatics programs PSORTb and PSLpred integrate various protein characteristics, such as evolutionary information of PSI-Blast, amino acid, and dipeptide composition, as well as 33 physicochemical properties. CELLO uses 4 types of sequence coding schemes: the amino acid composition, the di-peptide composition, the partitioned amino acid composition, and sequence composition based on the physicochemical properties of amino acids. The analysis of protein domains was performed using the following servers: PROSITE from the Swiss Institute of Bioinformatics (SIB) (http://prosite.expasy.org/prosite.html), and Conserved Domain Database (CDD) from the National Center for Biotechnology Information (NCBI) (http://www.ncbi.nlm.nih.gov/cdd/). The signal peptide in the amino acid sequences was predicted using these servers: InterPro from the European Bioinformatics Institute (http://www.ebi.ac.uk/interpro/); SignalP4.1 from the Center for Biological Sequence Analysis (http://www.cbs.dtu.dk/services/SignalP/), and Phobius from the Stockholm Bioinformatic Center (http://phobius.sbc.su.se/). Prediction of transmembrane helices was done using the TMHMM Server v. 2.0 from the Center for Biological Sequence Analysis (http://www.cbs.dtu.dk/services/TMHMM/), HMMTOP v 2.0 from the Research Centre for Natural Sciences (http://www.enzim.hu/hmmtop/html/submit.html) and Phobius programs.

### Statistical analysis

Data corresponding to adherence assays, and biofilm formation were analyzed using the Unpaired Student's *t*-test from the GraphPad software Inc. (La Jolla, CA, USA). The *p*-value used in the study was ≤0.005 as point of statistical significance.

## Results

### TCP, CFA/III, BFP, and CS21 share homologous proteins among them

**Figure 1:**
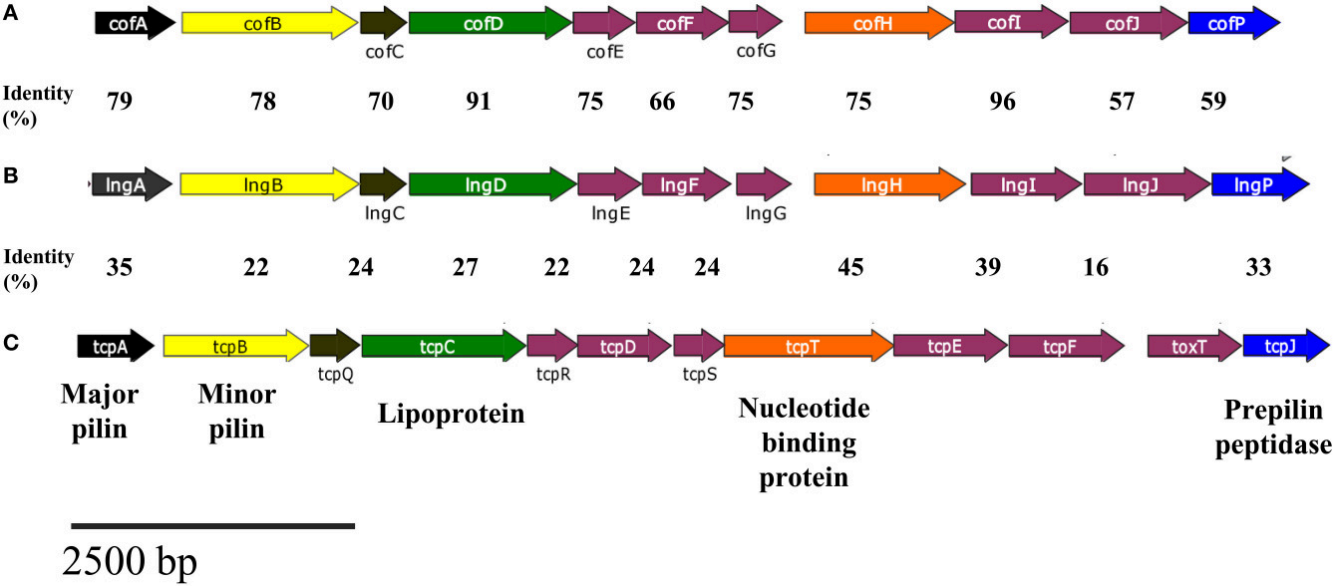
**Comparison of the genetic organizations of cluster related to type IV pili. (A)** *cof*, **(B)** *lng*, and **(C)** *tcp* gene clusters.The homologous of the *lngA, lngB, lngC, lngD, lngH*, and *lngP* genes in *cof* and *tcp* are indicated by the same color of the arrow. Amino acid sequences identities between CS21-CFA/III and CS21-TCP pili are shown in the figure.

TCP, CFA/III, BFP, and CS21 are type IVb pili, which biogenesis requires between 11 and 14 genes, encoded within the pilus operon (Roux et al., 2012). Several proteins involved in TCP, CFA/III, and BFP assembly are homologous with proteins encoded in the *lng* cluster (Gomez-Duarte et al., 2007; Roux et al., 2012). Comparison of the genetic organizations of *cof*, *lng*, and *tcp* gene clusters show a common genetic organization, and a similar size (Figure 1). The percentage of identity between CS21-CFA/III and CS21-TCP genes products are shown in Figure 1. LngA, CofA, and TcpA are the major pilin subunits and share a high percentage of identity to the amino acid level (Roux et al., 2012). LngB and CofB proteins have been proposed to be the minor pilin subunits of CS21 and CFA/III, respectively (Kolappan et al., 2015).

The gene and amino acid sequence similarity between CS21 components and those of CFA/III and TCP suggested the functions of the CS21 proteins. Additionally, we analyzed the amino acid sequences using predictive tools to gain further support for these assignments. Automatized prediction of bacterial protein subcellular localization is an important step to elucidate its function. Prediction analysis is based on the presence of a specific motif, protein characteristics, and homology to proteins with known localization (Yu et al., 2004, 2010; Bhasin et al., 2005). CELLO, PSLpred, and PSORTb were used to analyze the amino acid sequences of LngA, LngB, LngC, LngD, LngH, and LngP (**Table 4**). LngA, CofA, and TcpA proteins are the major pilin subunits of CS21, CFA/III, and TCP, respectively; their localization was predicted to be extracellular and experimentally has been demonstrated (Tables 3, 4). LngC and CofC are proteins with unknown function whose subcellular localization was predicted to be in the outer membrane (Table 4). On the other hand, TcpQ (the homolog of LngC with 24% of identity) is required for TcpC (secretin of TCP) stability and outer membrane localization (Bose and Taylor, 2005). LngD has a putative function as a multimeric outer membrane lipoprotein, and LngP as prepilin peptidase. LngD and LngP were predicted to be in the outer and inner membrane, respectively (Table 4). LngD homologs TcpC and BfpB were demonstrated experimentally to be lipoproteins and are located in the outer membrane (Ramer et al., 1996; Bose and Taylor, 2005). CofJ, TcpJ, and BfpP are homologous of LngP, with 59, 33, and 32% of identity, respectively. The homologous of LngP have been demonstrated to be prepilin peptidases of CofA, TcpA, and BfpA, respectively (Kaufman et al., 1991; Zhang et al., 1994; Taniguchi et al., 1999). Membrane protein topology prediction of LngP predicts inner membrane localization with 6 internal helices. LngH is predicted to be a nucleotide binding protein; its homolog TcpT (45% of identity) is the ATPase of TCP (Iredell and Manning, 1997). The subcellular localization of LngH was predicted into the cytoplasm. On the other hand, TcpT was localized in the inner membrane in a TcpR-dependent manner (Tripathi and Taylor, 2007).

**Table 3:** **Function of CS21, CFA/III, and TCP proteins**.

**Protein**	**Function**	**References**	**Protein**	**Function**	**References**	**Protein**	**Function**	**References**
LngA	Major pilin subunit	Girón et al., 1994	CofA	Major pilin subunit	Taniguchi et al., 1994, 1995	TcpA	Major pilin subunit	Taylor et al., 1987
LngB	Minor pilin subunit	Kolappan et al., 2015	CofB	Minor pilin subunit	Kawahara et al., 2015; Kolappan et al., 2015; Kawahara et al., 2016	TcpB	Unknown	
				Initiate pilus assembly				
LngC	Unknown		CofC	Unknown		TcpQ	Required for proper localization of TcpC to the outer membrane	Bose and Taylor, 2005
LngD	Lipoprotein^*^	Gomez-Duarte et al., 2007	CofD	Lipoprotein^*^	Taniguchi et al., 2001	TcpC	Lipoprotein	Bose and Taylor, 2005
LngE	Unknown		CofE	Unknown		TcpR	Bitopic IM protein^*^	Tripathi and Taylor, 2007
LngF	Unknown		CofF	Unknown		TcpD	Bitopic IM protein^*^	Roux et al., 2012
LngG	Unknown		CofG	Unknown		TcpS	Unknown	
LngH	Nucleotide binding, twitching motility^*^	Gomez-Duarte et al., 2007	CofH	Nucleotide binding protein^*^	Taniguchi et al., 2001	TcpT	ATPase	Iredell and Manning, 1997
LngI	Unknown		CofI	Unknown		TcpE	Polytopic IM protein^*^	Roux et al., 2012
LngJ	ATPase, twitching motility^*^	Gomez-Duarte et al., 2007	CofJ	Putative colonization factor	Yuen et al., 2013	TcpF	Colonization factor	Kirn et al., 2003
LngP	Prepilin peptidase^*^	Gomez-Duarte et al., 2007	CofP	Prepilin peptidase	Taniguchi et al., 1999	TcpJ	Prepilin peptidase	Kaufman et al., 1991; LaPointe and Taylor, 2000

* *Predicted function*.

**Table 4:** **Subcellular localization of CS21 proteins**.

**Protein**	**Localization**						
	**Putative function**	**CELLO**	**Scale 0–5**	**PSLpred (Hybrid approach)**	**Scale 0–100%**	**PSORTb**	**Scale 0–10**
LngA	Major pilin subunit	Extracellular	2.641	Extracellular	53.1	Extracellular	9.71
LngB	Minor pilin subunit	Extracellular	2.973	Extracellular	71.1	Unknown	
LngC	Unknown	Outer membrane	2.329	Outer membrane	53.1	Unknown	
LngD	Outer membrane lipoprotein	Outer membrane	4.611	Outer membrane	98.1	Outer membrane	9.92
LngH	ATPase	Cytoplasmic	3.610	Cytoplasmic	98.1	Cytoplasmic	9.97
LngP	Prepilin peptidase	Inner membrane	4.744	Inner membrane	71.1	Inner membrane	10

### The LngB, LngC, LngD, LngH, and LngP proteins are essential for CS21-mediated adherence to HT-29 cells and bacterial self-aggregation

**Figure 2:**
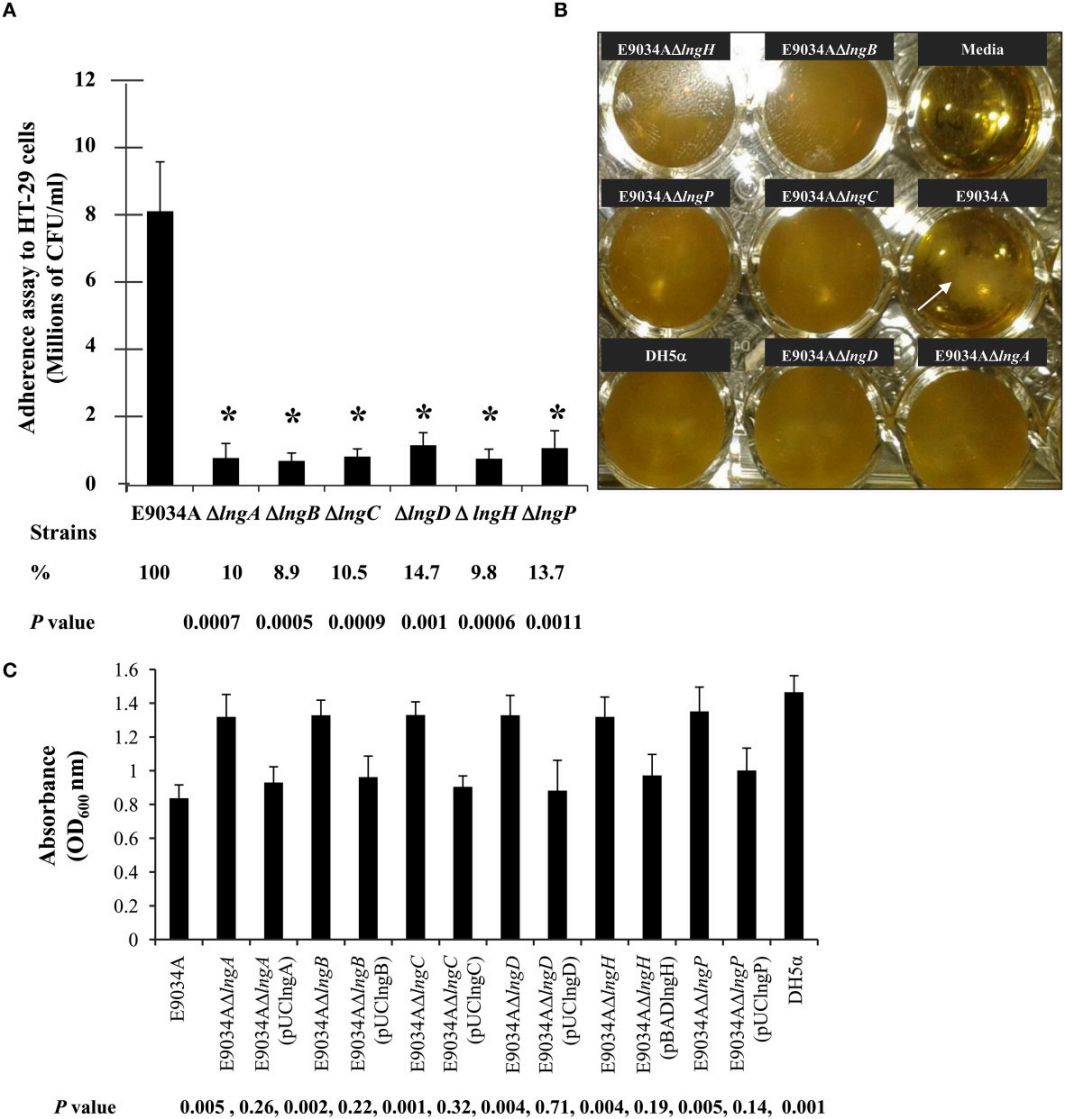
**Mutagenesis of ***lngA***, ***lngB***, ***lngC***, ***lngD***, ***lngH***, and ***lngP*** genes reduce CS21 adherence to HT-29 cells and bacterial self-aggregation. (A)** Quantitative analysis of HT-29 cells adherence by E9034A and isogenic mutants. **(B)** Self-aggregation assay in TB showing bacterial clumping in the E9034A strains (arrow) but not in the mutants examined. Non-inoculated media and the *E. coli* DH5α strain were used as negative controls. **(C)** Quantitative analysis of the self-aggregation assay. Absorbance at 600 nm from the supernatant of TB media with each mutant and complemented strains was plotted. These data represent the average of two experiments repeated on different days in triplicate. ^*^p ≤ 0.005 statistically significant difference compared to the wild-type strain.

**Figure 3:**
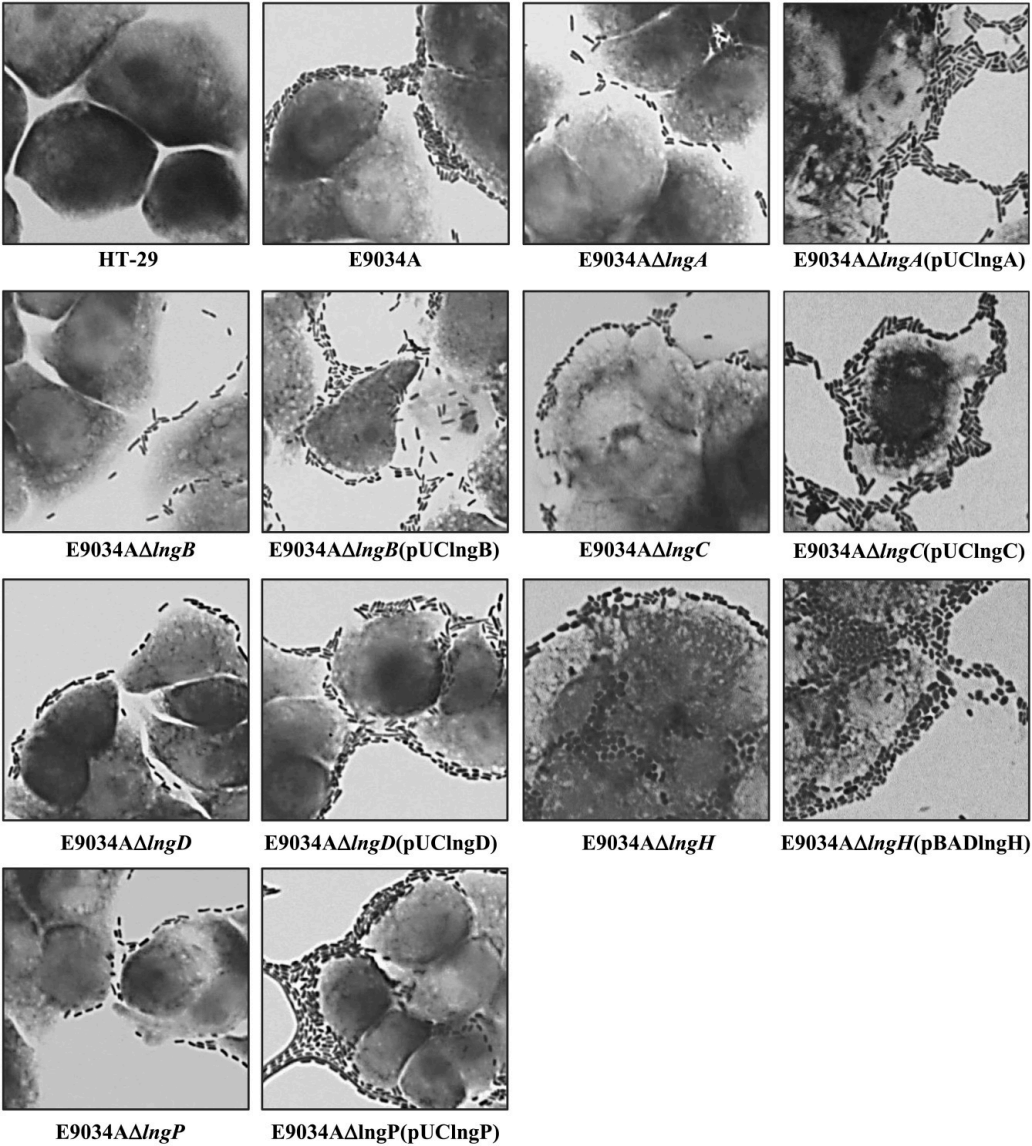
**Comparative analysis of HT-29 cells colonization by E9034A, isogenic mutants (E9034AΔ***lngA***, E9034AΔ***lngB***, E9034AΔ***lngC***, E9034AΔ***lngD***, E9034AΔ***lngH***, and E9034AΔ***lngP***) and complemented strains**. Giemsa staining of adherence assays on HT-29 cells showing differences in the number of bacteria attached to the cells. The complementation of the mutants by the addition of a wild-type copy of the mutant gene in *trans* restored the wild-type phenotype. Photographs taken at 100x.

CS21 is required to mediate human colonic cells colonization by ETEC CS21^+^ (Mazariego-Espinosa et al., 2010). We made deletions of the *lngB, lngC, lngD, lngH*, and *lngP* genes in the E9034A strain to evaluate their role in CS21 adherence. The growth rate was not affected in the mutants when compared with the wild-type strain (Supplemental Figure 1). Mutant strains showed 85–90% reduction on adherence to HT-29 cells compared to the wild-type strain (Figure 2). Bacterial self-aggregation mediated by CS21 causes bacterial clumping and media clearance. The mutants examined did not form clumps and sediment as the wild-type strain (Figure 2B). Complementation in *trans* of each mutant restored the adhesive properties and bacterial self-aggregation to wild type levels (Figures 2C, 3). All proteins tested in this study were involved in CS21 assembly and indirectly in adherence to HT-29 cells and bacterial self-aggregation.

### LngP is the prepilin peptidase of LngA in the E9034A strain

Type IV class b prepilins are processed by group I PilD-like prepilin peptidases (Ayers et al., 2010), resulting in the methylation of the first residue at the N-terminus of the mature protein and cleavage of the signal peptide (Hobbs and Mattick, 1993).

**Figure 4:**
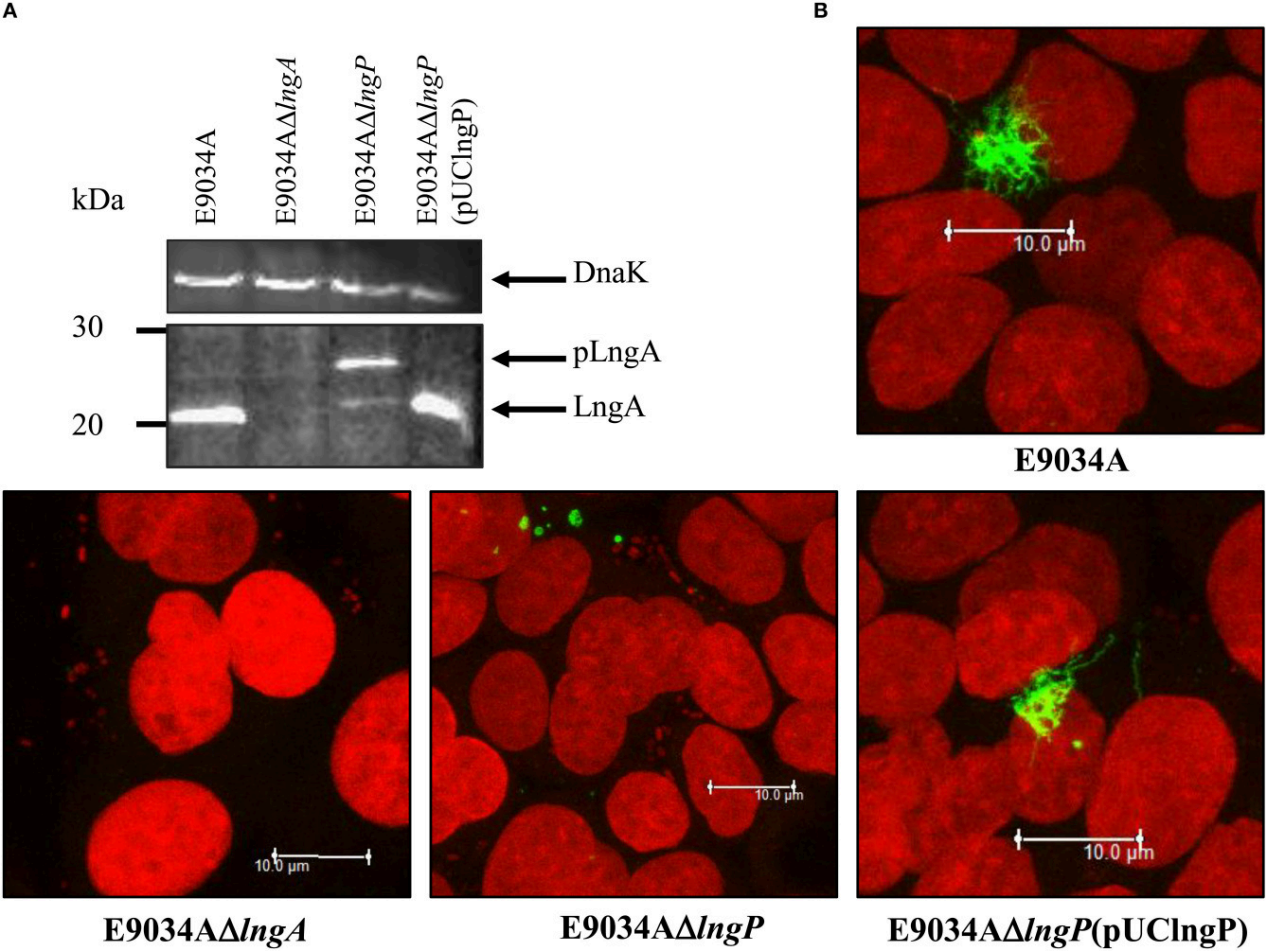
**LngP is the prepilin peptidase of LngA. (A)** Production of the LngA subunit detected by Western-blot of bacterial lysates, using a specific anti-CS21 serum. Anti-CS21 serum revealed a protein with a Mr (relative molecular mass) of 22-kDa in the E9034A strain, and one of 25-kDa in the *lngP* mutant, consistent with LngA (pilin) and pLngA (prepilin), respectively. **(B)** Phenotypic characterization by immunofluorescence assay of the E9034A, E9034AΔ*lngA*, E9034AΔ*lngP*, and E9034AΔ*lngP* (pUClngP) strains. The DNA was stained with propidium iodide (red) and CS21 structures were visualized with anti-IgG antibodies conjugated with Alexa fluor 488 (green). Photographs taken at 63x.

**Figure 5:**
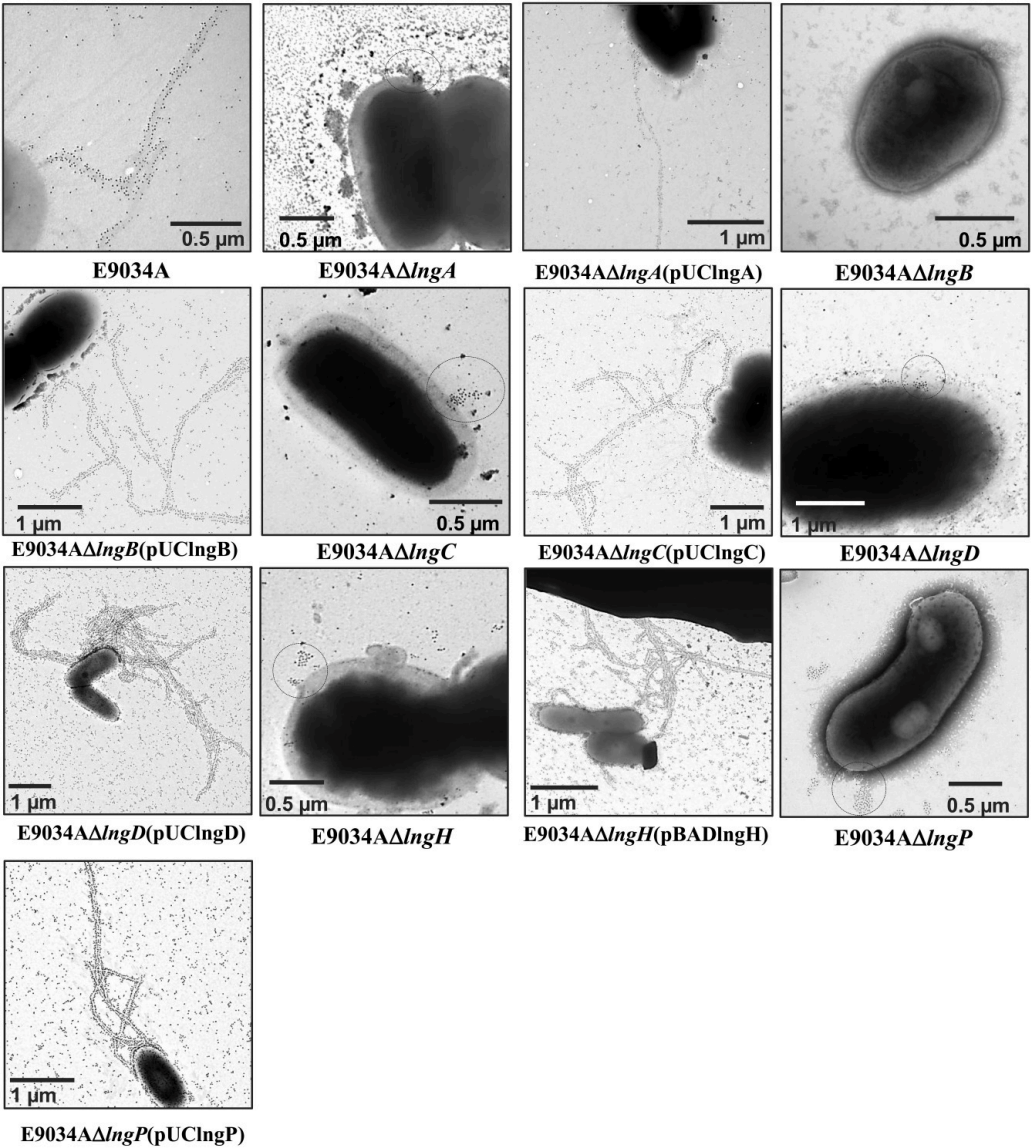
**Analysis of CS21 production in the ETEC E9034A strain and isogenic mutants by immunogold-labeling TEM**. CS21 production on the surface of the E9034A strain (arrow) was identified using rabbit anti-CS21 serum and goat anti-rabbit IgG gold conjugate. However, CS21 assembly was abolished in the *lngA, lngB, lngC, lngD, lngH*, and *lngP* mutants. On the surface of the *lngA, lngC, lngD, lngH*, and *lngP* mutants a cluster of gold particles were visualized by TEM, but not in the E9034AΔ*lngB* strain. The complementation of each mutant by the addition of a wild-type copy of the gene in *trans* restored the wild-type phenotype.

LngP protein is a putative prepilin peptidase with a conserved N-domain involved in the methyltransferase activity (CXXC and GXCXXC residues) and two highly conserved aspartate residues in the C-terminal domain that could be involved with the peptidase activity (Roux et al., 2012). LngA prepilin processing was affected in the E9034AΔ*lngP* strain, as demonstrated by an incomplete cleavage of LngA (Figure 4). The immunoblot showed two bands in the E9034AΔ*lngP* strain, a faint band of 22-kDa (pilin) and a stronger band of 25-kDa (prepilin), unlike the E9034A strain, which showed a single band of 22-kDa (pilin) (Figure 4). The deletion of the *lngP* gene showed a reduction in adherence of 86% compared to the wild-type strain (Figure 2). The complementation of E9034AΔ*lngP* strain in *trans* with the plasmid pUClngP restored the adherence levels and LngA cleavage (Figures 3, 4). This result suggests that LngP is the prepilin peptidase of LngA, and furthermore, that the E9034A strain has another prepilin peptidase capable of processing LngA prepilin, although with less efficiency. A minor proportion of LngA pilin identified in the E9034AΔ*lngP* strain by WB, suggests that this strain could be able to assemble CS21. To test this hypothesis we examined the E9034AΔ*lngP* and wild-type strain by immunofluorescence assay. Immunofluorescence assay demonstrated the presence of CS21 characteristic structures in the E9034A and E9034AΔ*lngP* (pUClngP) strains, but not in the E9034AΔ*lngA* strain (Figures 4B, 5). In the E9034AΔ*lngP* strain a faint reaction was observed by IF assays; however, the presence of CS21 structures was not evident by IEM (Figures 4B, 5).

### The anti-CS21 serum recognizes LngA and LngB proteins

To confirm CS21 assembly on the wild-type strain and mutants generated, immunogold-labeling TEM was carried out using specific anti-CS21 serum. Gold particles were found attached to long, polar, fimbrial structures on E9034A strain, typical features of CS21 pilus (Figure 5). In contrast, all the mutants tested by IEM no CS21 structures were observed (Figure 5). Clusters of gold particles on the cell surface were observed in the *lngA, lngC, lngD, lngH*, and *lngP* mutants instead, but not in the E9034AΔ*lngB* strain. The absence of gold particles in the E9034AΔ*lngB* strain was indicative of another antigen recognized by the anti-CS21 serum and also it showed that the secondary antibody did not label unspecifically the bacteria. These results suggest that the anti-CS21 serum could be recognizing both LngA and LngB proteins. Whole cell lysate of the E9034A strain and recombinant his-tag proteins of LngB and LngC were subjected to WB assay. The anti-CS21 serum recognized two proteins, one of ~22-kDa and other of ~60-kDa in the E9034A strain (Supplemental Figure 2A). The calculated molecular weight of the LngB protein is 57.04-kDa and the recombinant His-tag protein of 57.98-kDa. LngB-His and LngC-His recombinant proteins resolved by SDS-PAGE showed a molecular weight that correspond with the calculated molecular weight (Supplemental Figure 2B). Interestingly, the anti-CS21 serum recognizes the LngB-His protein, but not LngC-His protein; these results confirm that the anti-CS21 serum recognized LngA and LngB proteins (Supplemental Figures 2C,D). Notably, anti-CS21 serum by IEM recognizes both CS21 filament and tip as shown by the gold particles associated to the end of the filament (Supplemental Figures 2E,F).

### LngB is a minor pilin subunit of CS21

**Figure 6:**
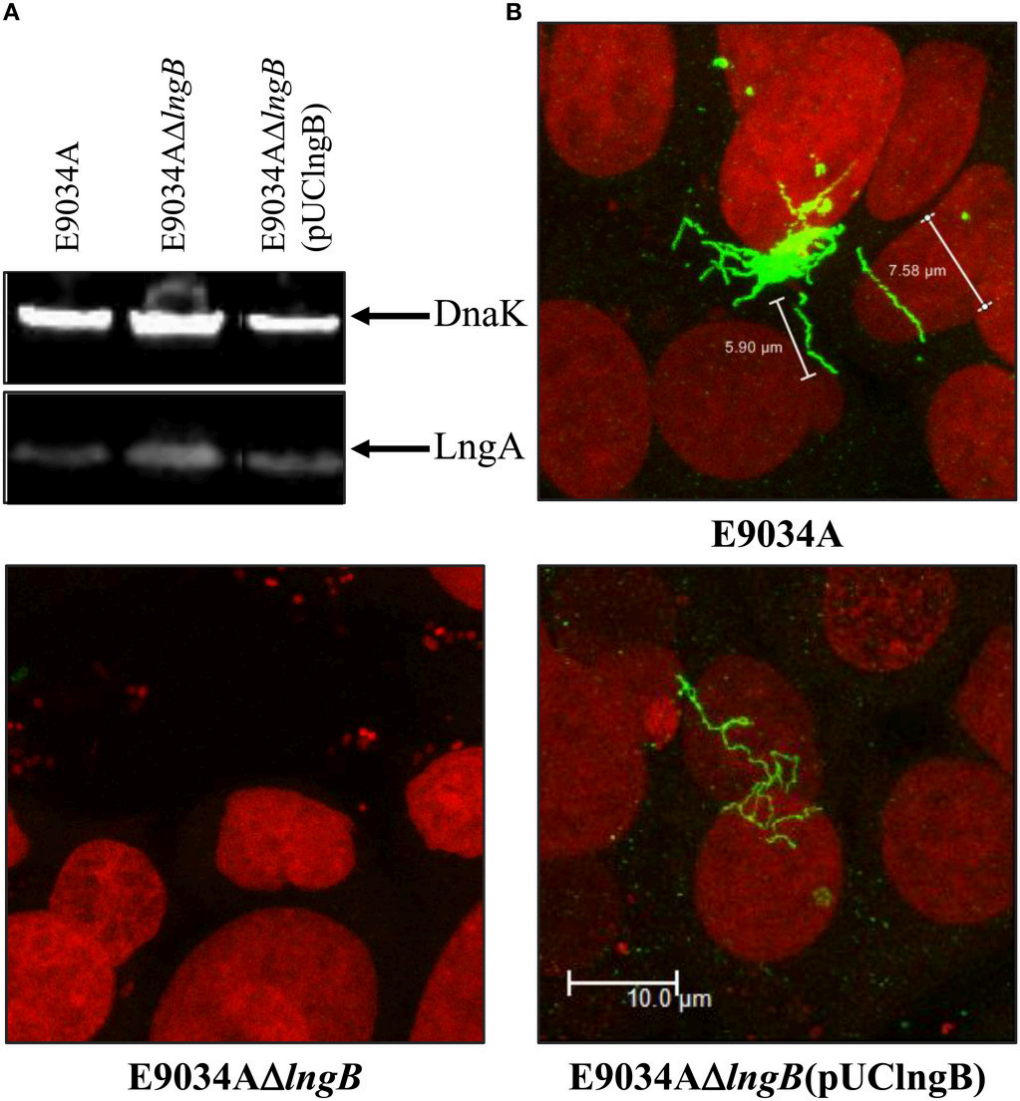
**Complementation of ***lngB*** gene in ***trans*** restored CS21 expression. (A)** Western-blot assay: whole protein extracts were separated by SDS-PAGE, transferred to PVDF membranes, and reacted with anti-CS21 serum and anti-DnaK antibodies. **(B)** Immunofluorescence assay: eukaryotic and bacterial DNA were stained with propidium iodide (red) and CS21 structures were visualized with anti-CS21 serum and goat anti-rabbit IgG antibodies conjugated with Alexa fluor 488 (green). The confocal microscopy micrographs were taken at a magnification of 63x.

**Figure 7:**
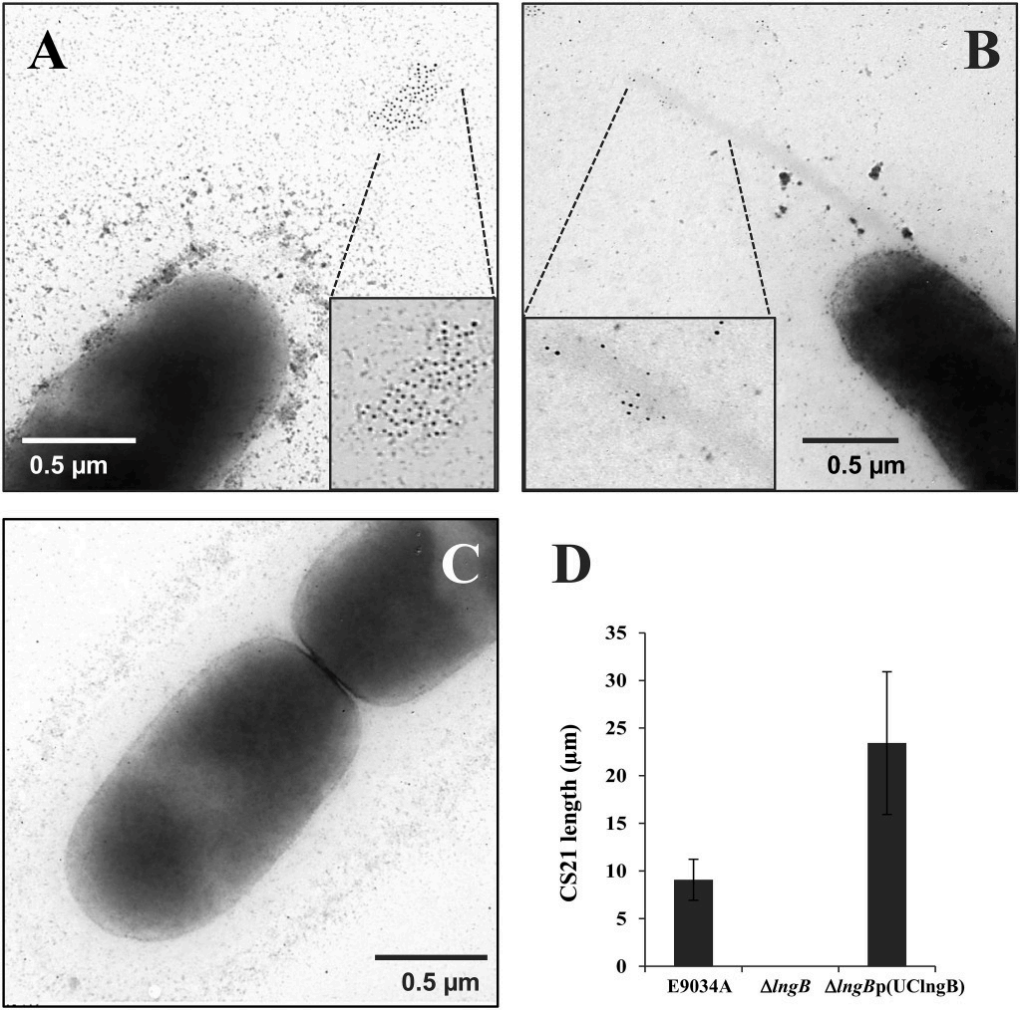
**LngB protein is a minor pilin subunit. (A,B)** Immuno-gold assay, using specific anti-LngB serum, showing that LngB is probably localized at the tip of CS21 in the E9034A strain. **(C)** As negative control the E9034AΔ*lngB* strain was used, absence of gold labeling was observed. **(D)** Measurements of CS21 length by confocal microscopy of the E9034A, E9034AΔ*lngB*, and E9034AΔ*lngB* (pUClngB) strains were plotted. This data was obtained from 20 different fields of two different experiments.

LngB protein is a minor pilin subunit, necessary to initiate the assembly of the CS21 pilus. CofB protein is the homologous of LngB protein in CFA/III and has been demonstrated that the C-terminal region of this protein is required to initiate the assembly of CFA/III pilus (Kolappan et al., 2015). In this study, we reproduced the results previously demonstrated by Kolappan et al. (2015). The *lngB* deletion did not affect LngA expression and processing as demonstrated by WB (Figure 6A). However, it disrupts CS21 assembly as shown by IEM and IF assay (Figures 5, 6B). These data suggest that LngB plays a role in LngA assembly, but not in LngA expression or processing. The subcellular localization of LngB was predicted to be extracellular, and this prediction correlates with the localization of the gold particles observed in the *lngA, lngC, lngD*, and *lngH* mutant strains, but not in the E9034AΔ*lngB* strain (Figure 7C). Immunogold assays using specific anti-LngB serum suggested that LngB is localized at the tip of CS21 in the E9034A strain (Figures 7A,B). ETEC E9034A strain produces more than one filament, the filaments intertwined forming hair like or bundles as described by Girón et al. (1991). We propose that the presence of the gold particles indicate the presence of various filaments associated with LngB at CS21 tip.

**Figure 8:**
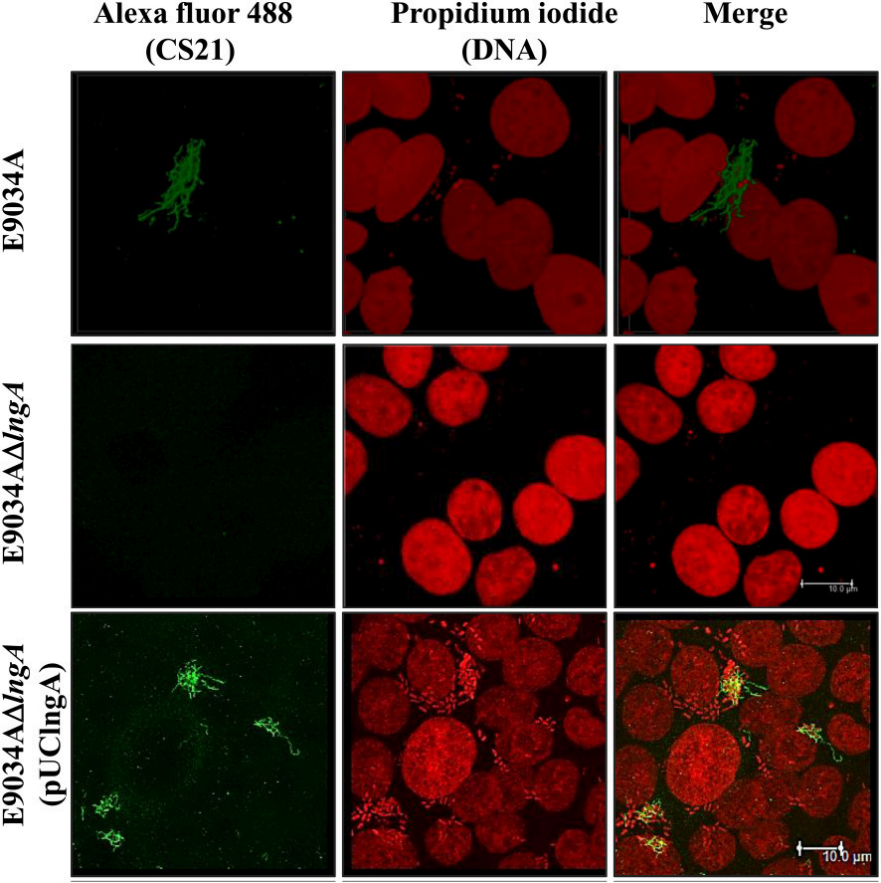
**Overexpression of LngA did not increase CS21 length**. Immunofluorescence and confocal microscopy assays: the DNA was visualized with propidium iodide (red), CS21 structures with anti-CS21 serum and polyclonal goat anti-rabbit IgG conjugated with Alexa fluor 488 (green). Images were taken at a magnification of 63x.

Genetic restoration of the *lngB* gene carried on pUClngB into the E9034AΔ*lngB* strain restored CS21 assembly as demonstrated by the presence of long, polar, and flexible structures (Figure 6B, Supplemental Figure 3). Immunofluorescence assays using anti-CS21 antibodies showed that the length of CS21 in the E9034AΔ*lngB* (pUCLngB) strain was longer than the structures visualized in the wild-type strain (Figure 6B). Quantitative analysis of confocal images allowed us to measure CS21 length; additionally, we found that the average length of CS21 from the E9034A strain was 9.01 ± 2.05 μm and the E9034AΔ*lngB* (pUCLngB) was 22.62 ± 6.77 μm (Figure 7D, Supplemental Figure 3). On the other hand, the E9034AΔ*lngA* (pUClngA) strain that was complemented using the same vector as the E9034AΔ*lngB* (pUClngB), did not show an CS21 length increase as the one observed for the E9034AΔ*lngB* (pUClngB) strain (Figure 8).

### LngC, LngD, and LngH proteins are required for CS21 assembly

LngC has an identity of 24, 20.3, and 70% with TcpQ, BfpG, and CofC, respectively (Figure 1), and is predominantly at the C-terminus, in which a conserved protein domain of the TcpQ family was found. The TcpQ protein is required for proper localization of TcpC protein (secretin) in the outer membrane of the bacteria (Bose and Taylor, 2005). On the other hand, the BfpG protein is required for the formation and/or stability of the multimer protein but not for the localization of BfpB protein (secretin) in the outer-membrane of the bacteria (Schmidt et al., 2001).

LngD and LngH are homologous to the outer membrane lipoprotein and nucleotide-binding protein, respectively, related to other type IV pili biogenesis apparatus (Roux et al., 2012). LngD has an identity of 17.6, 19.7, and 91% with TcpC, BfpB, and CofD respectively, and its C-terminus is homologous with the conserved domain of *pilus_B_mal_scr*, member of the secretin protein superfamily. On the other hand, LngH has an identity of 21.9, 44.7, and 75% with BfpD, TcpT, and CofH, respectively. LngH belongs to the P-loop NTPase superfamily, characterized by a conserved nucleotide phosphate-binding motif, also referred to as the Walker A motif [GxxxxGK(ST)] (Iyer et al., 2004).

**Figure 9:**
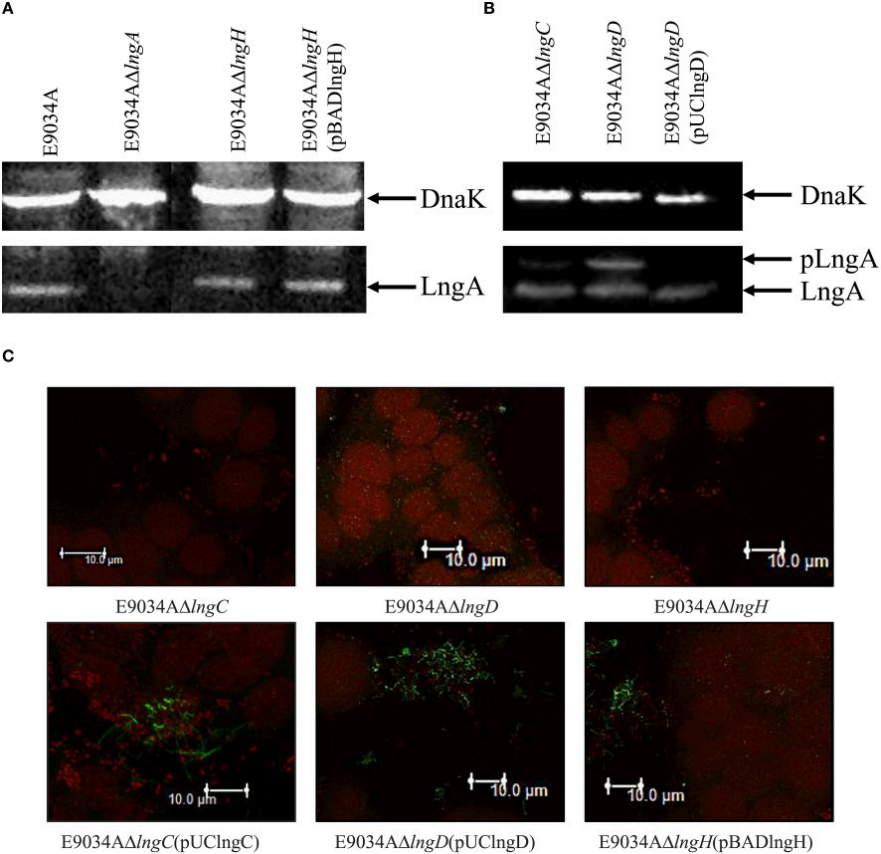
**The mutation of ***lngD*** and ***lngC*** disturbed LngA processing. (A,B)** Western-blot assay with whole protein extracts were separated by SDS-PAGE, transferred to PVDF membranes and reacted with anti-CS21 serum and anti-DnaK monoclonal antibodies. **(C)** Immunofluorescence assay: the DNA was stained with propidium iodide (red) and CS21 structures were visualized with anti-CS21 serum and polyclonal goat anti-rabbit IgG conjugated with Alexa fluor 488 (green). Photographs taken at 63x.

The deletion of *lngC* in the E9034A strain did not affect LngA expression, but the processing of LngA was incomplete as demonstrated by WB, with the recognition of two bands (prepilin and pilin), being the lower band (pilin) the one with higher intensity (Figure 9B). CS21 assembly was abolished in the E9034AΔ*lngC* strain as demonstrated by IEM and IF (Figures 5, 9C). Complementation of the *lngC* mutant by the addition of a wild-type copy gene in *trans* restores the wild-type phenotype (Figures 3, 5, 9). LngC has a signal peptide of 20 amino acids as predicted using the following softwares: SignalP4.1, Inter Pro and Phobius (Supplemental Figure 4A). The recombinant LngC-His protein was purified and subjected to SDS-PAGE. A double band of approximately 14 and 16 kDa was observed by Coomassie staining (Supplemental Figure 4B). Western blot analysis demonstrated a positive reaction with both bands using anti-His antibodies showing that LngC is processed (Supplemental Figure 4C).

Interestingly, the processing of LngA in the E9034AΔ*lngD* strain showed two bands by WB that correspond to prepilin (pLngA) and pilin (LngA); as a result of *lngD* gene deletion, CS21 pilus assembly was abolished (Figures 9B,C). In contrast, the E9034AΔ*lngH* strain did not affect the expression and processing of the LngA protein, but it affects the CS21 assembly (Figures 9A,C).

## Discussion

Type IV pili assembly is a process that requires the interaction of a complex biogenesis apparatus as described for the TCP, and BFP pili (Roux et al., 2012). Despite the homology that keep the different proteins involved in the biogenesis of CS21, TCP, BFP, and CFA/III, there are differences in the process of their assembly (Roux et al., 2012). Although, CS21 is related to TCP and BFP, the proteins involved in the assembly process have not yet been explored and the efforts only focused in elucidating how the LngA protein contributes in the colonization of ETEC to intestinal cells (Mazariego-Espinosa et al., 2010; Guevara et al., 2013).

A cluster of 14 genes in tandem (*lngR, lngS, lngT, lngA, lngB, lngC, lngD, lngE, lngF, lngG, lngH, lngI, lngJ*, and *lngP*) may be required for CS21 assembly (Gomez-Duarte et al., 2007). Additionally, proteins coded in the *lng* cluster are homologous to proteins involved in the biogenesis of TCP, BFP, and CFA/III (Gomez-Duarte et al., 2007; Roux et al., 2012). This homology was essential to propose the hypothesis about the protein's function coded in the *lng* cluster and required for CS21 assembly. Subcellular localization of CS21 proteins provides an important clue to their function. Protein subcellular localization by conventional techniques is costly and time consuming. Recently, many algorithms have been developed for subcellular localization prediction, based on neural networks and the supervised learning used in support vector machines (Reinhardt and Hubbard, 1998; Bodén and Hawkins, 2005; Matsuda et al., 2005). Analyses of subcellular localization and experimental data showed that LngA, BfpA and TcpA are extracellular proteins anchored to the inner membrane cell (Taylor et al., 1987; Girón et al., 1991, 1994). Despite the unknown function, LngC has been predicted to be an outer membrane protein according to bioinformatic analysis. We support this result based on the fact that the homologs proteins BfpG and TcpQ were designated as outer membrane proteins (Bose and Taylor, 2005; Daniel et al., 2006). Localization analysis of LngD (outer membrane protein) and LngP (inner membrane protein) correspond to BfpB/TcpC and BfpP/TcpJ, respectively. LngH and TcpT are homologous proteins, and their subcellular localization prediction was in the cytoplasm. TcpT and LngH lack any predicted transmembrane domain as it has been reported for other ATPases (Tripathi and Taylor, 2007). LngH has the typical Walker A (also known as the phosphate-binding loop) and B motif, which are associated with phosphate binding and ATP hydrolysis, respectively (Iyer et al., 2004). We hypothesize, that LngH is associated with CS21 assembly apparatus favored by the interactions with unknown proteins that are anchored to the inner membrane. LngB protein is a minor pilin subunit, whose subcellular localization was predicted to be extracellular protein by CELLO and PSLpred predictors; however, there is no experimental data about the subcellular localization of the LngB.

The prepilin proteins require processing by a prepilin peptidase, before translocation and oligomerization (Hobbs and Mattick, 1993; Strom and Lory, 1993). In this study, we demonstrated that the *lngP* gene coded for a prepilin peptidase and its absence shows a reduction in the LngA processing; however, deletion of the *lngP* gene did not completely abolish LngA processing. This data suggested the presence of another prepilin peptidase capable of processing the LngA protein in the E9034A strain. More than 30 genes in the *E. coli* K-12 genome sequence are homologous to genes of the general secretory pathway, involved in either secretion or type IV piliation including two with homology to genes coding for the prepilin peptidases *gspO* and *pppA* (Francetic and Pugsley, 1996; Francetic et al., 1998; Pugsley and Francetic, 1998). The *gspO* gene encodes a functional enzyme; however, its transcription levels are very low under laboratory conditions (Francetic and Pugsley, 1996). On the other hand, the *pppA* gene has been shown to code for a functional prepilin peptidase capable of processing typical prepilin peptidases substrates such as: prePulG (*Klebsiella oxytoca* type IV pre-pseudopilin), prePilE (*Neisseria gonorrhoeae* type IV prepilin), and prePpdD or preHcpA (enterohemorrhagic *E. coli* type IV prepilin “HCP”; Whitchurch and Mattick, 1994; Francetic et al., 1998; Xicohtencatl-Cortes et al., 2007). Interestingly, the LngA protein was completely processed in the *E. coli* TOP10 strain harboring the *lngA* gene in *trans* (Supplemental Figure 5). This data suggested that *E. coli* TOP10 prepilin peptidase (probably PppA) is functional and capable of cleaving typical prepilin peptidase substrates. In contrast, LngA partial processing observed in the E9034AΔ*lngP* strain, suggests the presence of another prepilin peptidase, probably PppA. The PppA and/or GspO proteins in ETEC strain E9034A may be involved in the maturation of LngA; however, they could be expressed at low levels compared to *E. coli* TOP10. Interestingly, partial processing of LngA was not enough for assembly of CS21, as demonstrated by IF and IEM using anti-CS21 serum. A previous report mentioned that a 5-kb Bam HI restriction fragment from the pE9034A mega plasmid was sufficient for pilus assembly and bacterial aggregation in an *E. coli* strain K-12 (DH5αF′IQ) (Girón et al., 1994). The 5-kb Bam HI restriction fragment from the partial sequences of the pE9034A plasmid was analyzed and we found that the *lngR, lngS, lngT, lngA*, and *lngB* (truncated sequence with 1106 bp) genes are contained in this fragment. *E. coli* K-12 contains 16 genes at seven different loci in its chromosome that encode components of type IV piliation machinery (Sauvonnet et al., 2000). These studies suggest that other genes in the *E. coli* K-12 chromosome are coding to proteins of type IV pilus and could replace the functions of the remaining CS21 proteins in the *E. coli* K-12 (DH5αF′IQ) but not in the *E. coli* E9034A strain.

The *lngA, lngB, lngC, lngD, lngH*, and *lngP* deleted strains showed a significant reduction in adherence to HT-29 cells, due to CS21 assembly failure. Gold particles evidenced the presence of long, polar, and fimbrial structures emerging from the E9034A strain surface, when reacted against anti-CS21 serum. However, *lngA, lngC, lngD, lngH*, and *lngP* deleted strains, showed a cluster of gold particles on the bacterial surface, but no CS21 labeled structures. Interestingly, a total lack of gold particles in the *lngB* gene deleted strain suggests that the anti-CS21 serum recognizes LngA and LngB proteins. Detection of LngA and LngB by the anti-CS21 serum could be the explanation for denser clusters in the *lngP* mutant strain than the rest of the mutants examined. To identify LngB localization, we generated a specific anti-LngB serum and showed for first time an extracellular localization for the LngB protein.

In this study, the mutation of *lngB* gene did not affect LngA expression and processing, but abolished CS21 assembly, and overexpression of LngB increased CS21 length in the E9034AΔ*lngB* (pUClngB) strain. In contrast, Kolappan et al. (2015) found that LngB overexpression did not affect CS21 assembly. Additionally, the three-dimensional structure of the CofB protein (homologous to LngB) determined by crystallography allowed to propose a model where CofB initiate and control the filament growth of CFA/III (Kolappan et al., 2015). Docking of CofB into CFA/III pilus filament model suggests a tip localization and is consistent with CofB's role as an initiator of pilus assembly (Kolappan et al., 2015). LngB protein is a minor pilin subunit, which subcellular localization by informatics analysis, homology with CofB and IEM images suggested is extracellular and probably at the tip of CS21. Future experiments will be conducted to determine if LngB is an adhesin of CS21.

The proteins coded by *lngC* or *lngD* genes might have an important role on pili basal apparatus assembly. We propose that the LngA prepilin, observed in the E9034AΔ*lngD* strain, causes an allosteric inactivation of LngP (prepilin peptidase) due to accumulation of LngA in the periplasm by the absence of the LngD (outer membrane secretin). The presence of unprocessed LngA in the E9034AΔ*lngC* strain suggests that LngC could be required for LngD stability and/or outer membrane localization. TcpQ, the homolog of LngC is required for TcpC (secretin of TCP) stability and outer membrane localization (Bose and Taylor, 2005). Deletion of *lngC* or *lngD* genes affected LngA processing, and thus, CS21 assembly, this effect is similar to their homologs in TCP. In this study, we showed that purified LngC is cleaved by a peptidase, probably LngP, as determined by a double band observed by Coomassie staining. LngC has 20.3% identity with BfpG, and the localization of BfpG to the outer membrane is dependent on BfpB (homolog of LngD; Daniel et al., 2006). The mutation of *bfpB* or *bfpG* genes does not affect BfpA expression or processing, but inhibits BFP biogenesis (Anantha et al., 2000). Interestingly, the purified BfpG-His protein migrates on acrylamide gels as a doublet. The authors propose that the mature BfpG is not a lipoprotein, and showed that purified BfpG exists in two forms after cleavage at either of two typical signal peptidase I sites, as determined by amino-terminal acid sequencing (Daniel et al., 2006). Similarly, the purified LngC-His protein migrates on acrylamide gels as a doublet; however, it was predicted that the LngC has a single cleavage site and the molecular weight from upper and lower band matches the molecular weight of the immature (16.29-kDa) and mature (13.99-kDa) protein, respectively.

**Figure 10:**
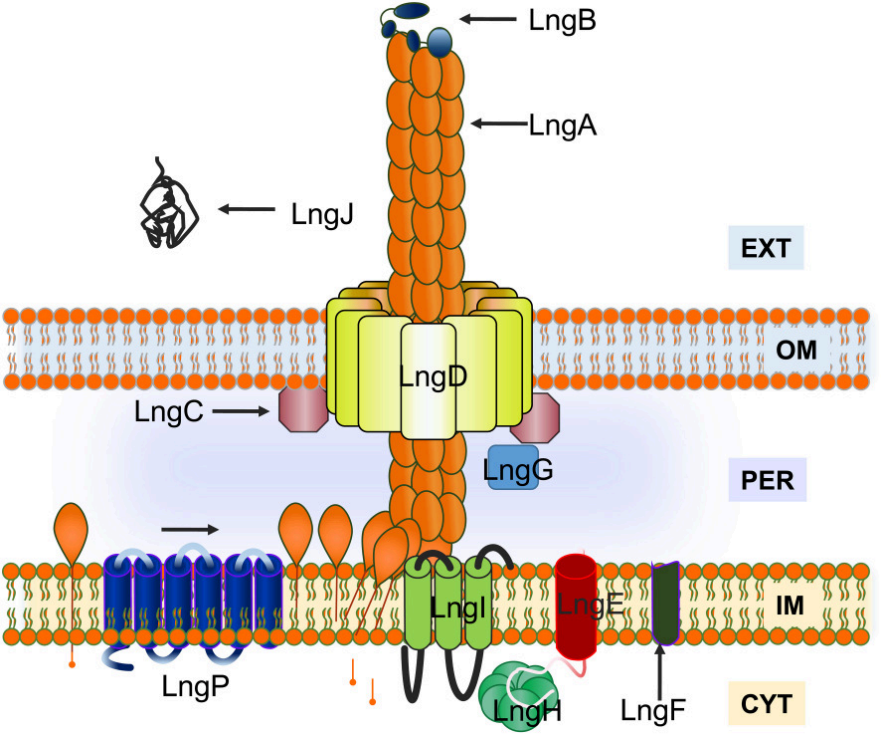
**Model for CS21 assembly in enterotoxigenic ***Escherichia coli*****. The LngA subunits oligomerize to form CS21. LngA subunits are synthesized as prepilins, with an N-terminal charged leader peptide, and processed by a prepilin peptidase “LngP.” LngB is a minor pilin subunit. Inner membrane (IM), outer membrane (OM), periplasm (PER), cytoplasm (CYT), and extracellular space (EXT) are indicated on the figure.

Type IVb pili have a genetic organization that required proteins coded by 12–14 genes clustered into operons (Roux et al., 2012). The proteins coded by genes in the *lng* operon have homology with proteins involved in TCP and BFP assembly. This homology was a tool to understand the assembly process of the CS21 pilus. In this study, it was demonstrated that LngA, LngB, LngC, LngD, LngH, and LngP proteins are essential to CS21 pilus assembly and the deletion of the genes that code for these proteins affect the bacterial self-aggregation and adherence phenotypes of E9034A strain. The assembly of type IVb pili requires various proteins forming the piliation assembly machinery into inner and outer membrane subcomplexes. A model for CS21 assembly was proposed based on proteins localization prediction, homology to other characterized proteins, identification of motifs, and *lngA, lngB, lngC, lngD, lngH*, and *lngP* genes deletions (Figure 10). LngA is the major pilin subunit that oligomerizes forming the filament. The N-terminal helices for the pilin subunits are bound to the inner membrane and are incorporate into the growing pilus from the inner membrane. LngP is an inner membrane prepilin peptidase that process LngA prepilin. LngB protein is a minor pilin subunit. LngC was predicted to be localized in the outer membrane, and based on its homology with TcpQ of TCP, could be required for LngD stability and/or outer membrane localization. The C-terminal of LngD has a region with homology to the conserved protein domain of the secretin superfamily, which is used by type IV pilus to translocate pilin subunits and macromolecules across the outer membrane. A nucleotide-binding protein is required to provide energy to the assembly of CS21. The *lngH* gene, encodes a putative ATPase, “LngH.” The remaining *lngR, lngS, lngT, lngE, lngF, lngG, lngI*, and *lngJ* genes were not included in this model, but are currently being studied. In conclusion, the model proposed for the CS21 assembly in this study is similar to the models for other type IV pili as TCP and CFA/III.

## Author contributions

Designed and conceived the experiments: ZS, JX. Performed the experiments: ZS, KE, and VR. Analyzed the data: ZS, BG, and JX. Contributed reagents/materials/analysis tools: BG, AC, GP, EL, JA, SO, RH, CE, and JX. Wrote and reviewed the manuscript: ZS, GR, AC, VR, KE, MG, BG and JX.

### Conflict of interest statement

The authors declare that the research was conducted in the absence of any commercial or financial relationships that could be construed as a potential conflict of interest.
