# Corrigendum: Effects of *lng* Mutations on LngA Expression, Processing, and CS21 Assembly in Enterotoxigenic *Escherichia coli* E9034A

**DOI:** 10.3389/fmicb.2017.00026

**Published:** 2017-01-20

**Authors:** Zeus Saldaña-Ahuactzi, Gerardo E. Rodea, Ariadnna Cruz-Córdova, Viridiana Rodríguez-Ramírez, Karina Espinosa-Mazariego, Martín A. González-Montalvo, Sara A. Ochoa, Bertha González-Pedrajo, Carlos A. Eslava-Campos, Edgar O. López-Villegas, Rigoberto Hernández-Castro, José Arellano-Galindo, Genaro Patiño-López, Juan Xicohtencatl-Cortes

**Affiliations:** ^1^Laboratorio de Investigación en Bacteriología Intestinal, Hospital Infantil de México Federico GómezCiudad de México, Mexico; ^2^Instituto de Fisiología Celular at the Universidad Nacional Autónoma de MéxicoCiudad de México, Mexico; ^3^Departamento de Genética Molecular, Universidad Nacional Autónoma de MéxicoCiudad de México, Mexico; ^4^Departamento de Salud Pública, Facultad de Medicina, Universidad Nacional Autónoma de MéxicoCiudad de México, Mexico; ^5^Laboratorio Central de Microscopía, Departamento de Investigación-SEPI, Instituto Politecnico NacionalCiudad de México, Mexico; ^6^Departamento de Ecología de Agentes Patógenos, Hospital General “Dr. Manuel Gea González,”Ciudad de México, Mexico; ^7^Departamento de Infectología, Hospital Infantil de México Federico GómezCiudad de México, Mexico; ^8^Laboratorio de Investigación en Inmunología y Proteómica, Hospital Infantil de México Federico GómezCiudad de México, Mexico

**Keywords:** ETEC, CS21, biogenesis, type IV pilus, adherence to intestinal cells, pilus

In the original article, we neglected to thank our sponsors. This has been rectified below. The authors apologize for this oversight. This error does not change the scientific conclusions of the article in any way.

## Funding

This work was supported by grant number CONACYT 133451 and public Federal Funds HIM/2008/051, HIM/2011/070, HIM/2012/033, HIM/2014/016, HIM/2015/034, and HIM/2013/061 from the Hospital Infantil de México Federico Gómez.

### Conflict of interest statement

The authors declare that the research was conducted in the absence of any commercial or financial relationships that could be construed as a potential conflict of interest.

