# Supplementary figures and images for: Effects of lng Mutations on LngA Expression, Processing, and CS21 Assembly in Enterotoxigenic Escherichia coli E9034A
Source: Front Microbiol. 2016 Aug 3;7:1201. doi: 10.3389/fmicb.2016.01201 (PMC4971541; doi:10.3389/fmicb.2016.01201)

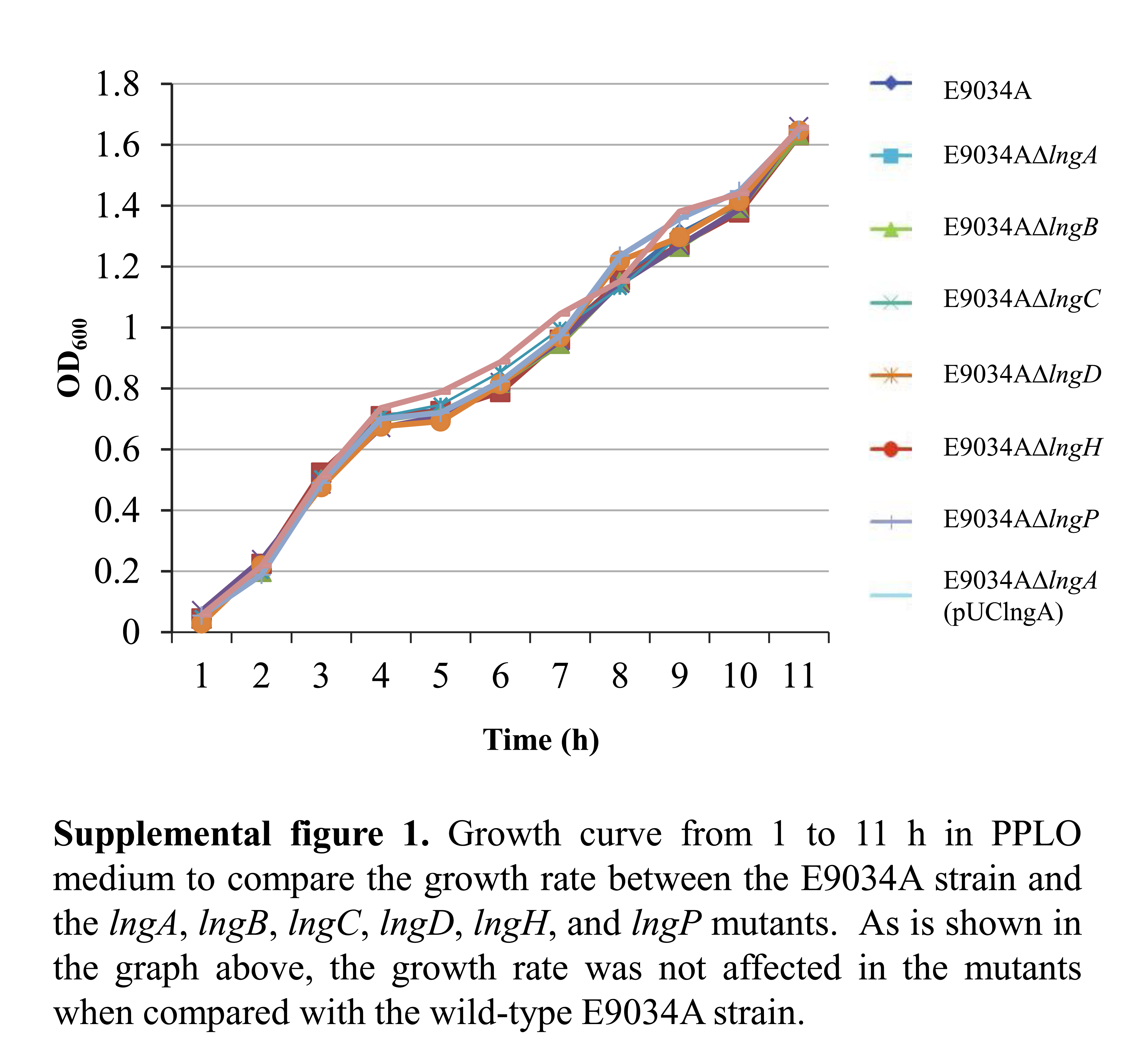

Supplement: Supplementary file 1 [file Image1.TIF]

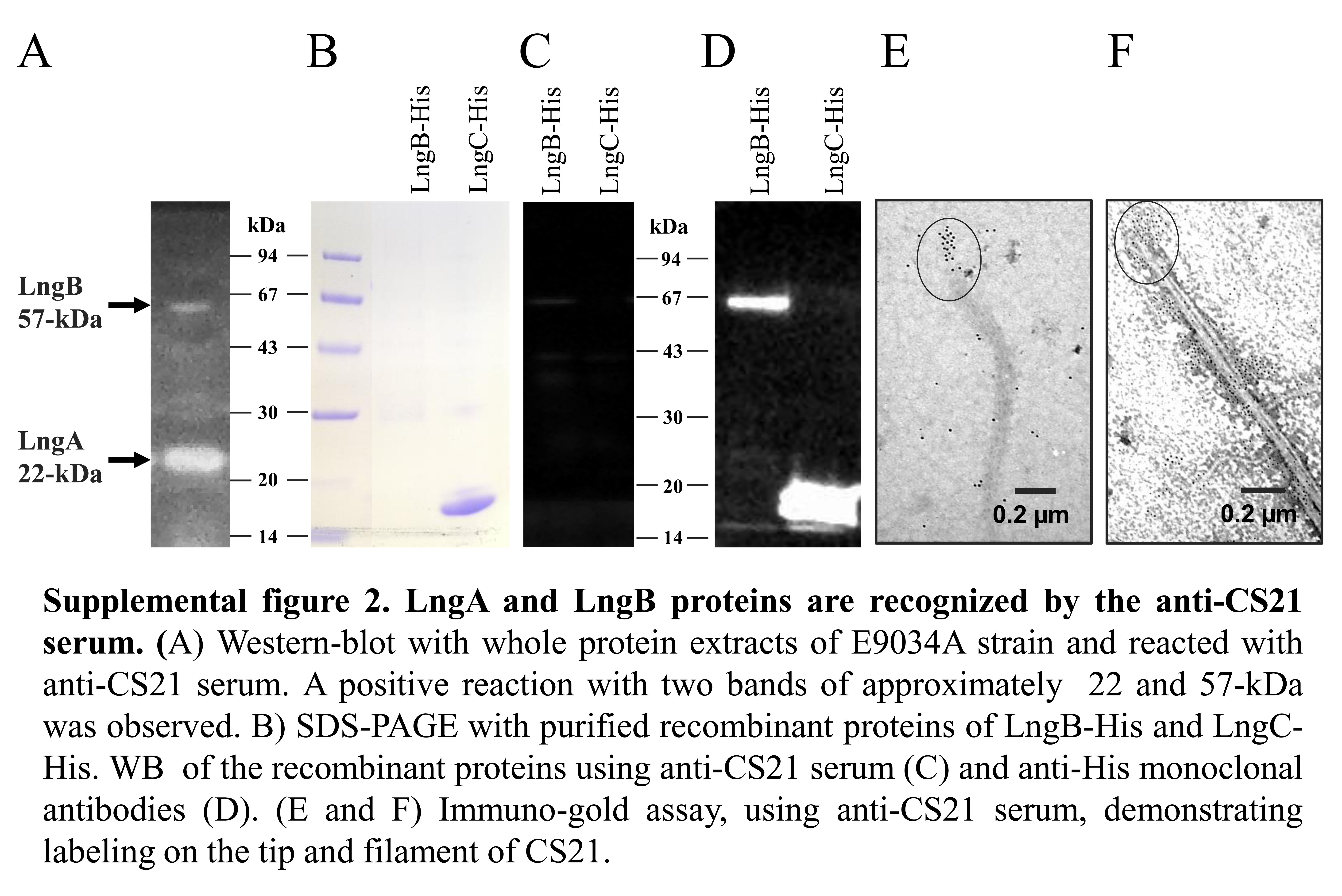

Supplement: Supplementary file 2 [file Image2.TIF]

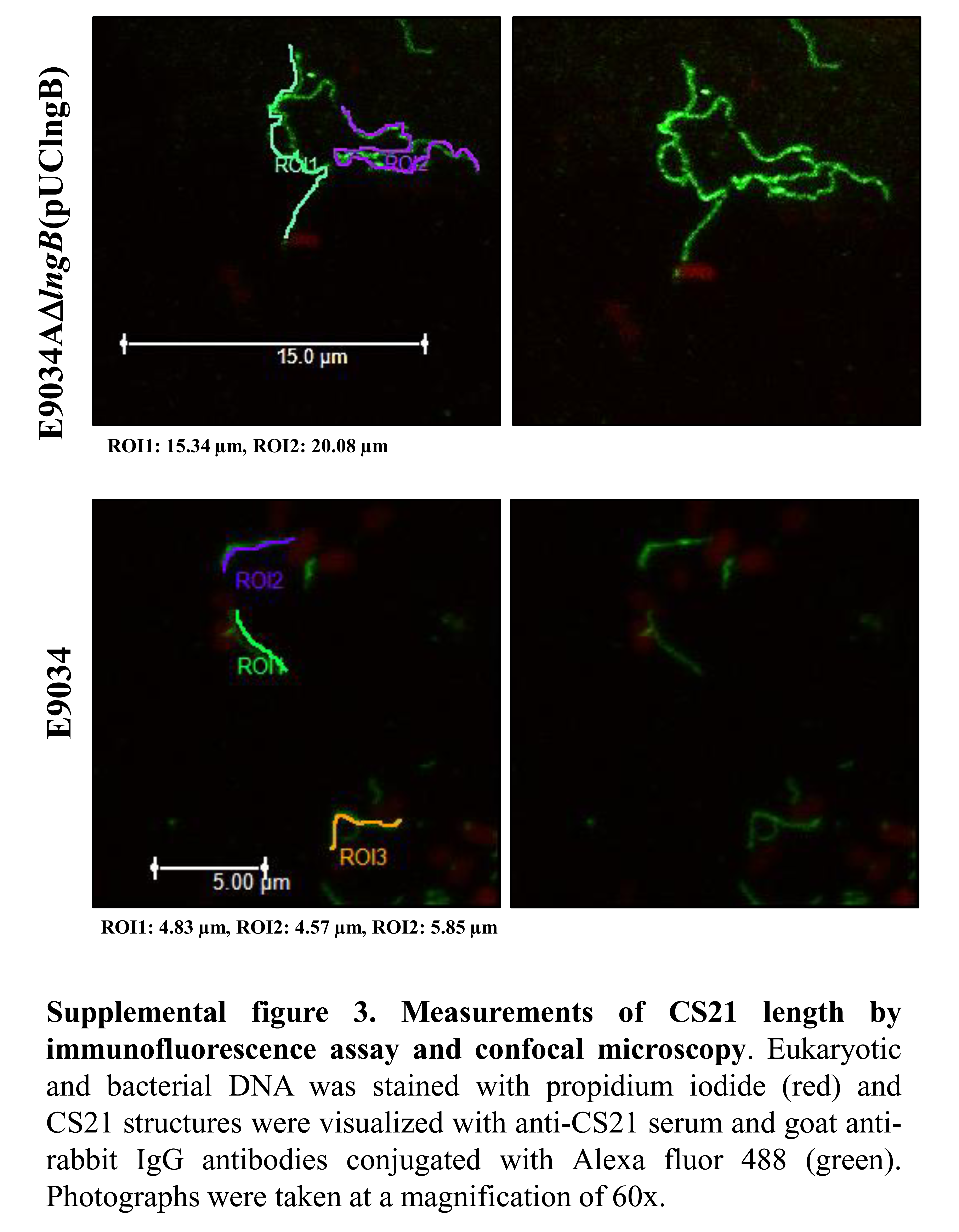

Supplement: Supplementary file 3 [file Image3.TIF]

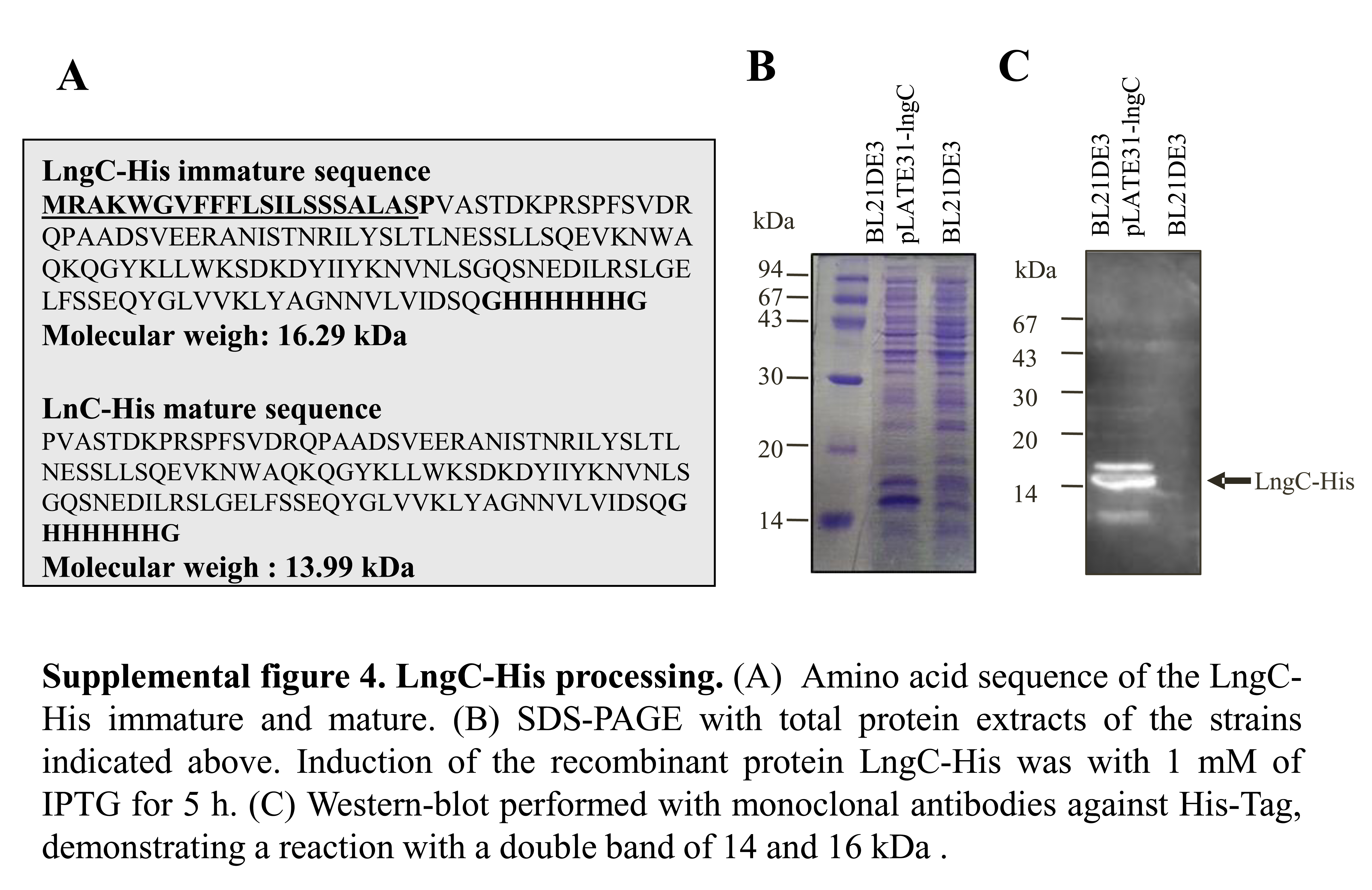

Supplement: Supplementary file 4 [file Image4.TIF]

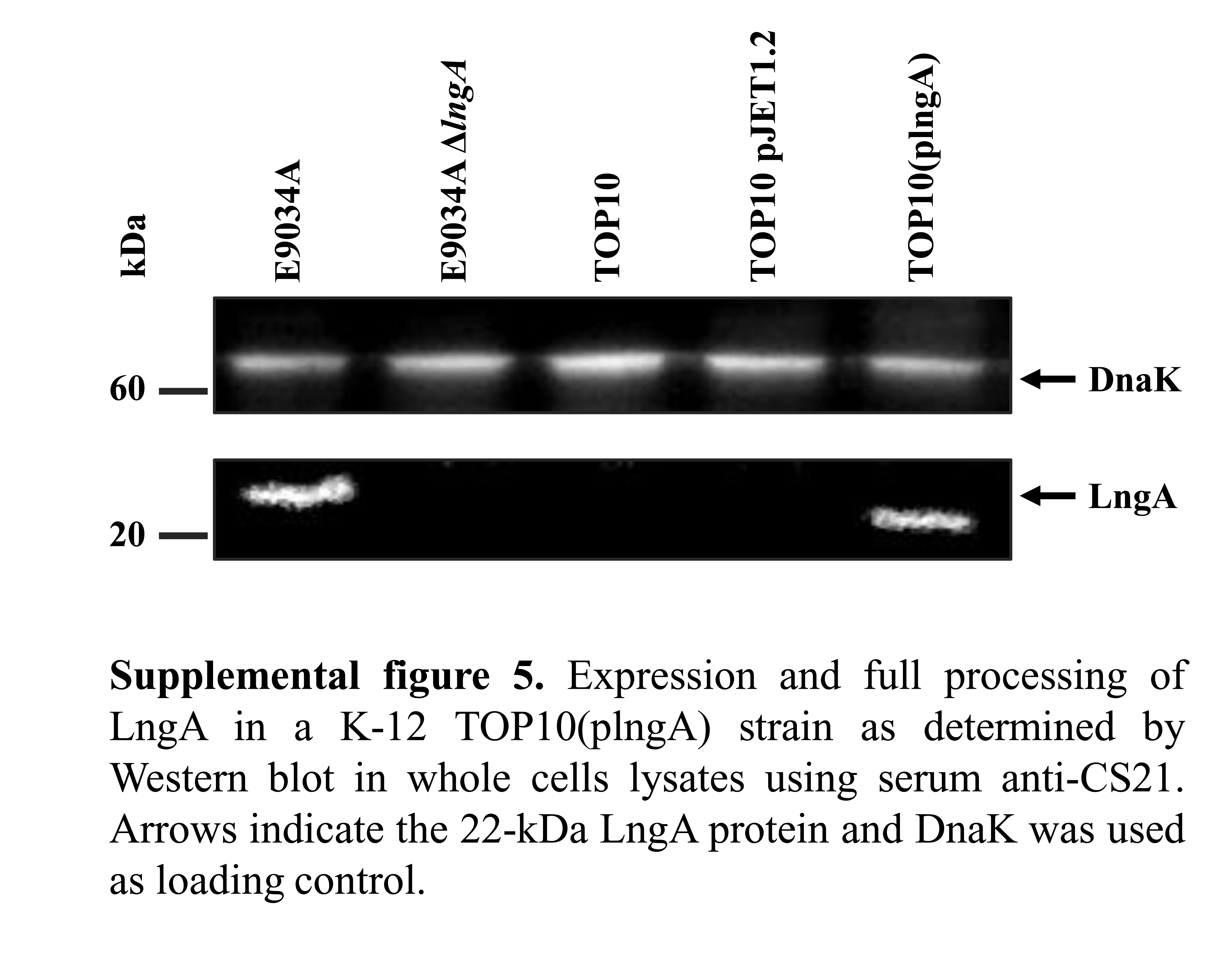

Supplement: Supplementary file 5 [file Image5.TIF]
